# Supplementary material for: Randomised trials conducted using cohorts: a scoping review
Source: BMJ Open. 2024 Mar 8;14(3):e075601. doi: 10.1136/bmjopen-2023-075601 (PMC10928784; doi:10.1136/bmjopen-2023-075601)
Supplement: Supplementary data [file bmjopen-2023-075601supp001.pdf]

Electronic search strategies

Adapted from the published protocol(24). Searches were run in both MEDLINE and Cochrane Methodology Register simultaneously. As an example, in the cohort search, lines 1-11 are the MEDLINE search and lines 12-15 are tailored for the Cochrane Methodology Register. The final lines of each search isolate the records from each database, combine them so duplicate records can be removed, then isolate the remaining records so they can be downloaded and imported into Reference Manager using customized import filters.

Searches for RCTs embedded in Cohorts

1. (cohort adj5 (randomi#ed adj5 trial*)).ab,kf,ti.

2. (cohort adj5 RCT*).ab,kf,ti.

3. (cohort adj5 controlled trial*).ab,kf,ti.

4. (cmRCT or Cohort Multiple Randomised Controlled Trial*).ab,kf,ti.

5. or/1-4

6. cohort.af.

7. (embed* adj8 randomi#ed).ab,kf,ti.

8. (embed* adj8 RCT*).ab,kf,ti.

9. (embed* adj8 controlled trial*).ab,kf,ti.

10. or/7-9

11. 6 and 10

12. (pragmatic adj5 RCT*).ab,kf,ti.

13. (pragmatic adj5 randomi#ed).ab,kf,ti.

14. (pragmatic adj5 controlled trial*).ab,kf,ti.

15. or/12-14

16. 6 and 15

17. 5 or 11 or 16

18. (meta analy* or metaanaly* or metanaly* or systematic review*).af.

19. 17 not 18

20. limit 19 to yr="2007 - 2018"

21. ((Cohort* and (random* or RCT)) or cmRCT).ti,ab,kw.

22. limit 21 to yr="2007 - 2018"

23. 20 use medall

24. 22 use clcmr

25. 23 or 24
26. remove duplicates from 25
27. 26 use medall
28. 26 use clcmr
