# Supplementary material for: Randomised trials conducted using cohorts: a scoping review
Source: BMJ Open. 2024 Mar 8;14(3):e075601. doi: 10.1136/bmjopen-2023-075601 (PMC10928784; doi:10.1136/bmjopen-2023-075601)
Supplement: Supplementary data [file bmjopen-2023-075601supp002.pdf]

Inclusion/Exclusion criteria for studies

Adapted from protocol for a scoping review to support development of a CONSORT extension for randomised controlled trials using cohorts and routinely collected health data(24).

Exclude: The publication is NOT reporting trials conducted using cohorts, protocols for RCTs that will be conducted using cohorts, describing a cohort intended to be used or has been used to conduct RCTs or methodological papers describing the use of cohorts. If the study involves non-human subjects it will be excluded.

Include: The publication describes an RCT in which the cohort is used or will be used to identify trial participants. The publication describes a cohort used to identify trial participants.

Include: The publication describes an RCT in which the cohort is used or will be used to ascertain health outcomes. The publication describes a cohort used to ascertain health outcomes.

Include: The methodological papers describe the use of cohorts for RCTs.
