# Supplementary material for: Randomised trials conducted using cohorts: a scoping review
Source: BMJ Open. 2024 Mar 8;14(3):e075601. doi: 10.1136/bmjopen-2023-075601 (PMC10928784; doi:10.1136/bmjopen-2023-075601)
Supplement: Author's manuscript [file bmjopen-2023-075601.draft_revisions.pdf]

BMJ Open

BMJ Open is committed to open peer review. As part of this commitment we make the peer review history of every article we publish publicly available.

When an article is published we post the peer reviewers' comments and the authors' responses online. We also post the versions of the paper that were used during peer review. These are the versions that the peer review comments apply to.

The versions of the paper that follow are the versions that were submitted during the peer review process. They are not the versions of record or the final published versions. They should not be cited or distributed as the published version of this manuscript.

BMJ Open is an open access journal and the full, final, typeset and author-corrected version of record of the manuscript is available on our site with no access controls, subscription charges or pay-per-view fees (http://bmjopen.bmj.com).

If you have any questions on BMJ Open's open peer review process please email
info.bmjopen@bmj.com

Randomised trials conducted using cohorts: A scoping review

Journal:	*BMJ Open*
Manuscript ID	bmjopen-2023-075601
Article Type:	Original research
Date Submitted by the Author:	12-May-2023

Complete List of Authors:	Nickolls, Beverley; Queen Mary University of London, Centre for Evaluation and Methods, Wolfson Institute of Population Health<br>Relton, Clare ; Queen Mary University of London, Centre for Evaluation and Methods, Wolfson Institute of Population Health<br>Hemkens, Lars; University Hospital Basel and University of Basel, Research Center for Clinical Neuroimmunology and Neuroscience Basel (RC2NB); University Hospital Basel and University of Basel, Basel,Switzerland, Department of Clinical Research<br>Zwarenstein, Merrick; Western University, Department of Family Medicine; Institute for Clinical Evaluative Sciences<br>Eldridge, Sandra; Queen Mary University of London, Centre for Evaluation and Methods, Wolfson Institute of Population Health<br>McCall, Stephen; National Perinatal Epidemiology Unit Clinical Trials Unit, Nuffield Department of Population Health, University of Oxford; Center for Research on Population and Health, Faculty of Health Sciences, American University of Beirut<br>Griffin, Xavier; Queen Mary University of London, Centre for Evaluation and Methods, Wolfson Institute of Population Health<br>Sohanpal, Ratna; Queen Mary University of London, Centre for Evaluation and Methods, Wolfson Institute of Population Health<br>Verkooijen, Helena; University Medical Centre Utrecht; University of Utrecht<br>Maguire, Jonathon; University of Toronto Institute of Health Policy Management and Evaluation<br>McCord, Kimberly; Independent Researcher

Keywords:	Clinical Trial, Randomized Controlled Trial, STATISTICS & RESEARCH METHODS, Patient-Centered Care

SCHOLARONE™
Manuscripts

I, the Submitting Author has the right to grant and does grant on behalf of all authors of the Work (as defined in the below author licence), an exclusive licence and/or a non-exclusive licence for contributions from authors who are: i) UK Crown employees; ii) where BMJ has agreed a CC-BY licence shall apply, and/or iii) in accordance with the terms applicable for US Federal Government officers or employees acting as part of their official duties; on a worldwide, perpetual, irrevocable, royalty-free basis to BMJ Publishing Group Ltd ("BMJ") its licensees and where the relevant Journal is co-owned by BMJ to the co-owners of the Journal, to publish the Work in this journal and any other BMJ products and to exploit all rights, as set out in our licence.

The Submitting Author accepts and understands that any supply made under these terms is made by BMJ to the Submitting Author unless you are acting as an employee on behalf of your employer or a postgraduate student of an affiliated institution which is paying any applicable article publishing charge ("APC") for Open Access articles. Where the Submitting Author wishes to make the Work available on an Open Access basis (and intends to pay the relevant APC), the terms of reuse of such Open Access shall be governed by a Creative Commons licence – details of these licences and which Creative Commons licence will apply to this Work are set out in our licence referred to above.

Other than as permitted in any relevant BMJ Author's Self Archiving Policies, I confirm this Work has not been accepted for publication elsewhere, is not being considered for publication elsewhere and does not duplicate material already published. I confirm all authors consent to publication of this Work and authorise the granting of this licence.

For peer review only

Randomised trials conducted using cohorts: A scoping review

Beverley Nickolls1

Clare Relton1

Lars G. Hemkens2,3,4,5

Merrick Zwarenstein6,7

Sandra Eldridge1

Stephen J. McCall8,9

Xavier Griffin1

Ratna Sohanpal1

Helena M. Verkooijen10,11

Jonathon Maguire12

Kimberly A. Mc Cord- De laco13

1. Centre for Evaluation and Methods, Wolfson Institute of Population Health, Queen Mary University of London, UK
2. Research Center for Clinical Neuroimmunology and Neuroscience Basel (RC2NB), University Hospital Basel and University of Basel, Basel, Switzerland
3. Department of Clinical Research, University Hospital Basel and University of Basel, Basel,Switzerland
4. Meta-Research Innovation Center at Stanford (METRICS), Stanford University, Stanford, California, USA
5. Meta-Research Innovation Center Berlin (METRIC-B), Berlin Institute of Health, Berlin, Germany
6. Department of Family Medicine, Western University, London, Canada
7. Institute for Clinical Evaluative Sciences, Toronto, Canada
8. National Perinatal Epidemiology Unit Clinical Trials Unit, Nuffield Department of Population Health, University of Oxford, Oxford, UK
9. Center for Research on Population and Health, Faculty of Health Sciences, American University of Beirut, Ras Beirut, Lebanon;
10. University Medical Center Utrecht, Utrecht, the Netherlands
11. University of Utrecht, Utrecht, the Netherlands
12. Institute of Health Policy, Management and Evaluation, University of Toronto, Canada
13. Independent researcher

Address for Correspondence: Beverley Nickolls, PhD student, Centre for Evaluation and Methods, Wolfson Institute of Population Health, Queen Mary University of London, UK
Email: b.nickolls@qmul.ac.uk

For peer review only - http://bmjopen.bmj.com/site/about/guidelines.xhtml

For peer review only

Acknowledgements:We thank Margaret Sampson, MLIS, PhD, AHIP (Children's Hospital of Eastern Ontario, Ottawa, Canada) for developing the search strategies on behalf of the CONSORT team. We thank Dr Philippa Fibert, St Mary's University, Twickenham, London for her help in screening publications for inclusion.

Abstract

Introduction: Cohort studies generate and collect longitudinal data for a variety of research purposes. Randomized controlled trials (RCTs) increasingly use cohort studies as data infrastructures to help identify and recruit trial participants and assess outcomes.

Objective: To examine the extent, range and nature of research using cohorts for RCTs and describe the varied definitions and conceptual boundaries for RCTs using cohorts.

Design: Scoping review

Data sources: Searches were undertaken in January 2021 in MEDLINE (Ovid) and EBM Reviews—Cochrane Methodology Registry (Final issue, third Quarter 2012).

Eligibility criteria: Reports published between January 2007 and December 2021 of (a) cohorts used or planned to be used, to conduct RCTs, or (b) RCTs which use cohorts to recruit participants and/or collect trial outcomes, or (c) methodological studies discussing the use of cohorts for RCTs.

Data extraction and synthesis: Data were extracted on condition being studied, age group, setting, country/continent, intervention(s) and comparators planned or received, unit of randomisation, timing of randomisation, approach to informed consent, study design and terminology.

Results: A total of 175 full text articles were assessed for eligibility. We identified 61 protocols, 9 descriptions of stand-alone cohorts intended to be used for future RCTs, 39 RCTs using cohorts and 34 methodological papers.

The use and scope of this approach is growing. The thematics of study are far-ranging, including population health, oncology, mental and behavioural disorders, and musculoskeletal conditions.

Authors reported this approach can lead to more efficient recruitment more representative samples and lessen disappointment bias and cross-overs.

Conclusion: This review outlines the development of cohorts to conduct RCTs including the range of use and innovative changes and adaptations. Inconsistencies in the use of terminology and concepts are highlighted. Guidance now needs to be developed to support the design and reporting of RCTs conducted using cohorts.

(297/300 words)

Strengths and Limitations

For peer review only - http://bmjopen.bmj.com/site/about/guidelines.xhtml

- The study's methodological strengths include the use of the CONSORT- ROUTINE Extension search strategy to update the scope of work taking place using cohorts.
- Another strength is the overview of methodological writing on trials using cohorts including how informed consent procedures are operationalized.
- The review was limited by the amount of detail available on some important design features e.g. how informed consent processes were operationalised in different clinical populations.
- While we aimed to assess reporting and terminology, the identification of our sample relied on reporting and terminology.

INTRODUCTION

In medical research the term cohort designates a group of persons who have one or more characteristics in common (1). Cohort studies use longitudinal data obtained from a cohort population to investigate the epidemiology of diseases and establish assocations between explanatory factors and health outcomes. Randomised controlled trials (RCTs) create treatment groups through random allocation in order to assess the effects of interventions such as screening, surgery or drugs. Traditional RCTs are often expensive and time consuming; often rendering designs that are of limited utility including small sample sizes, low generalisability and lack of long-term outcomes. There is an increasing need for innovative research designs to overcome these shortcomings(2-9).

RCTs conducted within cohorts is an innovative approach which uses new or existing cohort infrastructures and cohort study participants to identify and recruit trial participants and collect outcome data, through the regular measurement of cohort outcomes and procedures(10, 11).

Cohorts data infrastructures generate and collect data for the purpose of research and RCTs can be conducted using the cohort infrastructure. RCTs can also use administrative data(12), Electronic Health Records(EHR)(13) and registry data infrastructures(14), i.e. the classic forms of routine data collection. In this scoping review we cover RCTs using research based cohorts, but not RCTs using routine, administrative and registry based data infrastructures which have been described elsewhere(12-15).

Compared with traditional RCTs there are several advantages to using cohorts to conduct RCTs. If an existing cohort study is used then the time needed for developing trial recruitment and data collection systems can be reduced. RCTs conducted within cohort studies may recruit a more generalisable sample of participants(16). Recruiting trial participants from a cohort provides information on those who decline to participate in the trial. The cohort can also provide ongoing

For peer review only - http://bmjopen.bmj.com/site/about/guidelines.xhtml

For peer review only

information as to the natural history of the condition and treatment as usual and collect outcomes well beyond the time horizon of many traditional RCTs.

There is variation in how this innovative approach to trial design is named and described; a commonly cited approach is the cohort multiple RCT (cmRCT) design(2), another frequent and more recent term is the Trials within Cohorts (TwiCs) design(11). In this design, participants enrol in an observational cohort study with regular outcome measurement. This provides a framework for the implementation of multiple RCTs. For each RCT embedded in the cohort, a random selection of RCT-eligible patients is contacted and offered the intervention. Outcomes of participants randomly allocated to the intervention group are compared to outcomes of RCT-eligible patients not randomly allocated to the intervention, who receive standard/usual care as defined within the cohort. The information provided to potential trial participants and the consents sought are ‘patient centred’. The processes in this design aim to replicate informed consent practices as they would be applied in clinical care, i.e. patients are only informed about an intervention if and when they will have access to the intervention(2).

There is variation in how informed consent is operationalised in trials using cohorts. Some RCTs using cohorts use a standard approach to informed consent. In the standard approach all potential trial participants are informed of all group allocations and also that group allocation will be determined by chance not choice. This information is provided before randomisation. Other trials conducted using cohorts take different approaches to how and when potential and actual participants are given information and their consent sought. A key feature of the TwiCs design is the staging of informed consent. The origins of this approach aimed to be ‘patient-centred’. The ‘staged-consent’ approach(17) further refines this by seeking explicit consent from everyone recruited at the cohort enrolment stage to not be informed in the future if they are randomized to the usual care control group of an RCT.

Timing of randomisation of eligible cohort participants varies. Many trials randomise at one moment in time, using a single-batch sampling approach in their closed or recruiting cohorts. Some use a sequential process whereby individual patients are randomised as soon as they become eligible -which may or may not coincide with diagnosis(18). Given the relative novelty of RCTs using cohorts and the increasing adoption of these designs (1), there is a need to understand how this approach is being used in health research. This scoping review supplements the development of the Consolidated Standards of Reporting Trials (CONSORT) extension for the reporting of randomised controlled trials conducted using cohorts and routinely collected data

For peer review only - http://bmjopen.bmj.com/site/about/guidelines.xhtml

(CONSORT-ROUTINE)(19), which was developed to guide the reporting of the unique characteristics of trials conducted using both research cohorts and three kinds of routinely collected data (EHR, registry, and administrative data)(20).

The primary aim of this review is to examine the extent, range and nature of research activity for RCTs conducted using cohorts. Given the heterogeneous terminology and overlapping design details that became obvious during the development of reporting guidelines(19), the secondary aim is to clarify working definitions and conceptual boundaries for RCTs using cohorts.

METHODS

The design and reporting of this review follows the PRISMA Extension for Scoping Reviews guidelines(21). The protocol is accessible at Open Science Framework: https://osf.io/ke8pw(22)

A scoping review is defined as 'a form of knowledge synthesis that addresses an exploratory research question aimed at mapping key concepts, types of evidence, and gaps in research related to a defined area or field by systematically searching, selecting, and synthesizing existing knowledge'(23).

Search strategy

The search strategy from the original scoping review that was conducted to support the development of the CONSORT Extension for Trials using Cohorts and Routinely Collected Health Data(20, 24) was updated to cover January 2007 to December 2021. The original search strategy was designed and conducted by an experienced research librarian familiar with knowledge synthesis related to research methods and reporting. This was in collaboration with the CONSORT project team and peer reviewed using the Peer Review of the Electronic Search Strategy (PRESS)(25).

Searches were performed to identify publications describing methodology, trial protocols and results from RCTs that were conducted using cohorts. Searches were undertaken in January 2022 in Ovid MEDLINE Epub Ahead of Print, In-Process & Other Non-Indexed Citations, Ovid MEDLINE Daily and Ovid MEDLINE and EBM Reviews—Cochrane Methodology Registry (Final issue, third Quarter 2012). Searches were conducted covering a fifteen year period from January 2007- December 2021 which allowed the identification of relatively recent RCTs that used cohorts (supplementary file 1). Ten subject experts within the field were contacted to identify new protocols for trials that are being planned and other publications that may have been missed.

For peer review only - http://bmjopen.bmj.com/site/about/guidelines.xhtml

The references were imported from the database into Refworks, duplicates removed, and then transferred into DistillerSR (Evidence Partners, Ottawa, Canada). A coding manual based on eligibility criteria was developed (Supplementary file 2) A pilot test of the coding manual was performed prior to the study's inception.

Titles and abstracts were screened independently by two reviewers. A liberal accelerated method was used to identify articles for inclusion for full-text review, where titles and abstracts are screened by one reviewer (BN) and excluded articles are screened by a second reviewer (CR)(26). Full texts were screened independently by two reviewers (BN and PF), and any dissonance was resolved by a third reviewer (CR). At the full-text screening, each reviewer indicated how the cohort was used (used for recruitment, ascertainment of outcomes, both recruitment and outcomes).

Eligibility criteria

Inclusion criteria: any report of cohorts or results of a) cohorts that are used (or planned to be used), to conduct RCTs, b) RCTs within cohorts which use cohorts to recruit participants and/or collect trial outcomes c) methodological studies discussing the use of cohorts for RCTs (Supplementary file 2).

Data extraction

Data were extracted into a Google Documents spreadsheet. For articles reporting primary research the following information was extracted: condition being studied using ISRCTN registry condition categories(27), age group, setting (primary/secondary care/ other), country/ continent, intervention(s) planned or received, unit of randomisation (individual/ cluster) and comparator.

We also extracted data relating to the use of the cohort (participant identification, collection of outcome data), the terminology used to describe the trial design approach, and the approach used to inform and seek consent. Data extraction was completed by a single reviewer (BN) and independently validated by a second reviewer (CR).

Analysis and clarity of reporting

The reporting of this review followed the guidelines of the Preferred Reporting Items for Systematic Reviews and Meta-Analyses extension for scoping reviews (PRISMA-ScR)(21). The results were reported as a mixture of descriptive numerical synthesis and narrative synthesis. The

For peer review only - http://bmjopen.bmj.com/site/about/guidelines.xhtml

protocols and results of cohorts and RCTs in cohorts are reported in the tabular analysis and in the narrative synthesis and the methodological articles are described through narrative synthesis of themes for history and development of the terminology and concepts.

RESULTS

The results of the search are presented in Figure 1. Of 2,628 potentially relevant citations, 143 were eligible of which 109 articles were suitable for both quantitative analysis and qualitative synthesis and 34 methodological articles for qualitative synthesis only.

Of the 61 protocols, the majority (62%, 38/61) described planned cohorts and RCTs together. 18% (11/61) were for protocols for cohorts and 20% (12/61) were for RCTs in cohorts.

Figure 1 PRISMA flowchart (uploaded separately)

1. Research activity for trials conducted using cohorts.

In total 109 publications were reviewed to assess the extent, range and nature of research activity for trials conducted using cohorts. This included 61 protocols reporting on planned cohorts and/or RCTs, 39 papers reporting RCT results and 9 papers reporting results of cohorts studies established with the facility to host future RCTs. Table 1 reports the characteristics of articles reporting protocols for cohort studies and RCTs in column 1 (green). Articles reporting the results of RCTs embedded in cohorts and the results of cohorts set up hosting RCTs are reported in columns 2 and 3. The references throughout give examples of publication in these three categories. Results are presented as absolute values and percentages.

Table 1: Characteristics of publications reporting protocols for cohorts and results of trials using cohorts.

	No (%) of PROTOCOLS of cohort studies +/- RCTs) (n/61)		No (%)of RESULTS of RCTs embedded in cohort studies (n/39)		No (%) of RESULTS of cohort studies set up to host future RCTs (n/ 9)	
	n=	%	n=	%	n=	%
**Use of Cohort**						
Participant identification	61	100%	39	100%	9	100%
Outcome data	54	89%	36	92%	9	100%

For peer review only - http://bmjopen.bmj.com/site/about/guidelines.xhtml

**Condition being studied**						
Signs & symptoms (population health)	21	34%	10	26%	4	44%
Musculoskeletal diseases	12	20%	8	21%	0	0
Mental and Behavioural disorders	12	20%	3	8%	0	0
Cancer	8	13%	3	8%	3	33%
Infections and infestations	4	7%	3	8%	0	0
Respiratory	4	7%	2	5%	0	0
Urological and genital diseases	3	5%	3	8%	0	0
Nutritional Metabolic, endocrine	2	3%	2	5%	0	0
Cardiovascular	1	2%	3	8%	0	0
Skin and connective tissue diseases	1	2%	0	0	1	11%
Injury, occupational diseases, poisoning	1	2%	0	0	0	0
Ear, nose and throat	0	0	0	0	1	11%
Pregnancy and childbirth	1	2%	5	13%	0	0
Oral health	0	0	2	5%	0	0
Other	0	0	1	3%	0	0
**Age group**						
Adults	46	75%	34	87%	7	78%
Children	9	15%	5	13%	1	11%
Both	6	10%	0	0	1	11%
**Setting**						
Secondary care	40	66%	22	56%	5	56%
Primary care	12	20%	12	31%	4	44%
Other	9	15%	5	13%	0	0
**Country/ Continent**						
Continental Europe	22	36%	15	38%	4	44%
United Kingdom and Ireland	21	34%	13	33%	3	33%
North America	7	11%	3	8%	1	11%
Australasia	5	8%	2	5%	1	11%
Asia	3	5%	3	8%	1	11%
South America	2	3%	0	0%	0	0
Africa	1	2%	3	8%	0	0
**Intervention (s)**						
Behaviour change	17	28%	13	33%	N/A	0
Surgical	13	21%	5	13%	N/A	0
Drug	4	7%	12	31%	N/A	0
Psychological approaches	9	15%	3	8%	N/A	0
Chemo-radiation	5	8%	2	5%	N/A	0
Complementary therapy	3	5%	4	10%	N/A	0
Physical/Manual therapy	2	3%	5	13%	N/A	0
Screening	2	3%	2	5%	N/A	0
Other	10	16%	5	13%	N/A	0
**Comparator**						
Usual care	50	82%	28	72%	7	78%
Placebo	6	10%	6	15%	0	0
Unclear/Other	5	8%	5	13%	2	22%

For peer review only - http://bmjopen.bmj.com/site/about/guidelines.xhtml

Use of Cohort

All included studies used cohorts to identify potential participants and almost all 91% (99/109) also used the cohort to obtain some or all outcome data. Of the 39 RCT results papers, almost all (92%, 36/39) described trials which had used cohorts for both recruitment and outcome measurement. Only three RCT results papers reported using cohorts just for recruitment.

Conditions being studied

This section describes the conditions being studied using ISRCTN registry condition categories(27).

The most common category was signs and symptoms (population health and risk prevention) covering almost a third of protocols (22/61) , a quarter of articles reporting trial results (10/39) and more than a third of cohort descriptions (4/9). Where there was also a specific disease/condition the study was also placed under this category. For example, a protocol for a population health Covid-19 cohort (28) was placed within both the signs and symptoms category and the respiratory category. A cohort that recruited in pregnancy was categorised as Pregnancy and Childbirth as well as Signs and Symptoms/Population Health(29).

Age groups

Most studies were of adults - 75% (46/61) of protocols, 87% of RCT results (34/39) and 78% of cohort descriptions (7/9). Studies of children included 13% (5/39) of RCT results, 15% of protocols (9/61) and 11% of cohorts (1/9). Only 10% of protocols (6/61) studied both adults and children. These included planned cohorts for both parents and children (29, 30) and a trial that straddled the child/adult threshold (31).

Settings

The most common setting was secondary care with 66% of protocols (40/61), 56% of RCTs (22/39) and 56% of cohorts (5/9). Primary care settings accounted for 20% of protocols (12/61), 31% of RCTs (12/39) and 44% (4/9) of cohorts. The remaining studies described RCTs 15% (9/61) and protocols 13% (5/39) in community(32) and educational(33)(34) settings.

For peer review only - http://bmjopen.bmj.com/site/about/guidelines.xhtml

Country/Continent

The majority of publications came from Continental Europe, with 36% (22/61) protocols, 38% (15/39) RCTs and 44% (4/9) stand-alone cohorts. This was closely followed by the United Kingdom and Ireland, with contributions from North America, Australasia, Asia, South America and Africa.

Interventions

The most common interventions studied were behaviour change with 28% (17/61) of protocols and 33% (13/39) of RCTs. Some population health based cohorts planned to identify interventions after the cohort study had started data collection and thus were unable to identify which interventions would be tested e.g.(29, 30, 35, 36)). Some interventions fell into two or more categories, either because it could be classed as both, for example behavioural change and psychological approaches e.g.(37) or because the trial combined different categories of interventions, e.g. complementary therapy and nutrition e.g.(38). Some interventions were were categorised as 'other' e.g. helmet therapy(39), cough treatment algorithms(40) and nurse-led medicine monitoring(41).

Comparators

Most studies included treatment as usual (TAU) comparators - 82% (50/61) of protocols, 72% (28/39) of RCTs and 78% (7/9) of cohorts as defined within the cohort. TAU is assumed to refer to unrestricted usual care with no intervention, restriction or standardisation placed on the clinician or recipient but this was not clarified in some studies. Placebo was used in 10% (6/61) of protocols(42, 43) and 15% (6/39) of RCTs(44-46). The 'other' category included an active comparator(47).

In several cohorts it was not stated what the trial comparators would be, e.g.(48) but as expected, all RCTs using the staged approach had a usual care comparator. In the results of stand-alone cohorts intended to be used for RCTs we categorised them as having a usual care comparator if the publication made reference to using a staged or person centred approach to informed consent.

2.Terminology and Conceptual Boundaries

In addition to publications reporting research activity (Table 1), there were 34 methodology papers (Table 2). These were analysed to explore the development of the terminology and concepts and contribute to the qualitative synthesis. This table shows the type of trial design,

For peer review only - http://bmjopen.bmj.com/site/about/guidelines.xhtml

approach to informed consent, terminology used to describe the type of trial design and timing of randomisation to an RCT. The protocols are reported together in column 1 and the results of the RCTs and cohorts for hosting RCTs are given in columns 2 and 3.

Table 2: Methodological Papers

Focus of Paper	Numbers (n/34)		First author and Reference
	n	%	
Cohort designs for clinical areas	6	18	Ahmed(49), Couwenberg(18), Gal(50), Heaven(51), Young-Afat(52) Zakrzewska(53)
CmRCT/TwiCs design	17	50	Bibby(54), Candlish(55), Clegg(56), Flory(57), Kim(58), Lambin(59), Pate(60), Relton(2, 11), Reeves(61) Richards(54), Van der Velden(62), Verkooijen(63), Verweij(64), Vickers(65) Viksveen(16), Young-Afat(17)
Learning Health Systems (using TwiCs)	1	3	Wouters(66)
Studies within a Trial (SWATs) using cohorts	10	30	Arundel(67), Cockayne(68), Cotterill(69), Boyd(70), Goodwin(71), Knapp(72), Ni(73), Loban(74), Maruani ((75) Wakabyashi(74),

Type of Trial Design

The majority of protocols and trials reports described individually randomised RCTs. Cluster trial designs were described in 8% (5/61) of protocols and trial reports (3/39) and stepped wedge designs in 3% (2/61) of protocols and 5% (2/39) of trial reports. Table 3 reports the characteristics of articles reporting protocols for cohort studies and RCTs in column 1 (green). Articles reporting the results of RCTs embedded in cohorts and the results of cohorts set up hosting RCTs are reported in columns 2 and 3. The references throughout give examples of publication in these three categories. Results are presented as absolute values and percentages.

Table 3:Terminology and Conceptual Boundaries in articles reporting protocols for cohorts, and results of trials using cohorts.

	No (%) of PROTOCOLS of cohort studies +/- trials (n/61)		No (%)of RESULTS of RCTs embedded in cohort studies (n/39)		No (%) of RESULTS of cohort studies set up to host future RCTs (n/9)	
	n=	%	n=	%	n=	%
**Trial design**						

Individually randomised	54	89%	34	87%	7	78%
Cluster randomised	5	8%	3	8%	0	0
Stepped wedge	2	3%	2	5%	0	0
Not known	0	0%	0	0	2	22%
**Informed consent approach**						
'Patient-centred'/'Staged-consent'	32	52%	12	31%	7	78%
'All Upfront' standard approach	19	31%	21	54%	0	0
Not described	2	3%	2	5%	0	0
Other	8	13%	4	10%	0	0
**Design terminology used**						
Cohort multiple RCT (cmRCT)	16	26%	6	15%	5	56%
Trials within Cohorts (TwiCs)	12	20%	5	13%	1	11%
Cohort embedded RCT	9	15%	12	31%	0	0
Cohort nested RCT	11	18%	4	10%	0	0
Other	13	21%	12	31%	3	33%
**Timing of randomisation to RCT**						
Sequential randomisation	34	56%	15	38%	0	0
Batch randomisation	20	33%	23	59%	0	0
Not stated/known	7	11%	1	3%	9	100%

Approach to Informed Consent

Some studies stated whether they were using either a 'patient-centred' or 'staged' approach to informed consent, either by using these terms or by citing Young-Afat(17) 'Staged consent' publication or Relton(2) when describing their approach.

Over half (52%) of protocols (32/61) and 31% (12/39) of trials reported using a staged approach. Studies not using this approach included cluster trials(34, 76-79).

Terminology

The term cohort multiple RCT (cmRCT) was used in 26% (16/61) of protocols and 15% (6/39) of RCTs. Trials within Cohorts (TwiCs) was used in 20% (12/61) of protocols and 13% (5/39) of trials. Cohort embedded RCT was used in 15% (9/61) of protocols and 31% (12/39) of trials. Other terms used include cohort nested RCT, cohort RCT and cohort study.

In relation to informed consent there were 68 publications that used or described 'patient-centred'or staged approaches. Publications were identified if they either a) used the term 'patient-centred' or 'staged' or cited Young-Afat(17) or Relton(2) when describing their approach. One study cites using a staged approach but does not use any terminology to describe the design of its future RCTs(80). The terminology used in the 67 remaining publications is reported in table 4.

For peer review only - http://bmjopen.bmj.com/site/about/guidelines.xhtml

Table 4 reports terminology of studies using staged informed consent in methodological publications and publications reporting research activity. This table shows the terminology for methological literature, protocols and RCTs using cohorts. The reference to each publication is given and the colour index is given below the table. The terms cmRCT, cohort embedded and cohort nested are given from 2010 when the patient-centred approach to IC was first described and the term TwiCs from 2016 when it was first coined in the literature. Cohort-embedded and cohort-nested are given together with the codes 'em' for embedded and 'n' for nested.

For peer review only

For peer review only - http://bmjopen.bmj.com/site/about/guidelines.xhtml

Table 4:Terminology used to describe trial designs for RCTs with a patient-centred/staged approach to IC.

Methodological literature (n=24) Protocols (n=31). RCTs using cohorts (n=12)												
Trial design/ Year	2010	2011	2012	2013	2014	2015	2016	2017	2018	2019	2020	2021
cmRCT	(2)			(63)	(54)	(56, 59)	(17, 51, 53, 57, 60)	(16, 55, 81)	(61)			
		(82),(35)	(83)	(84)	(85, 86)	(31, 87)	(88, 89)	(90)	(91, 92)			
			(93)		(94)			(95, 96)	(97-99)			
TwiCs								(11)	(58, 61, 100)		(18, 66)	(52, 64, 101, 102)
							(29, 103)		(33)	(104-106)	(30, 107-110)	(111-113)
									(37, 114)	(38)	(115, 116)	
Cohort embedded/ nested RCT					(49)							
				(117)		(118)		(40)			(119)	

Table 4 Index:
Methodological literature (n=24)
Protocol (n=31)
RCTs using cohorts (n=12)

For peer review only - http://bmjopen.bmj.com/site/about/guidelines.xhtml

From 2018 onwards there is a noticeable shift in the terminology used in protocols, with the majority of protocols describing Trials within Cohorts (TwiCs) a name that was coined at the 2016 Ethics of TwiCs Symposium(11). This shift in terminology was also seen in the methodological literature where 100% (9/9) papers identified from 2018-2021 refer to the design as Trials within Cohorts, with one paper using both terms TwiCs and cmRCT(61).

Timing of randomisation

Fifity six percent (34/61) of the protocols and 38% (15/39) of the RCTs stated that would or had used sequential randomisation compared to batch randomisation which was used by 33% (20/61) of protocols and 59% (23/39). Timing of randomisation was not clear in 11% of protocols and 3% of RCTs.

DISCUSSION

Three main areas emerged from the narrative synthesis of themes arising in the studies and methodological literature: commentary on efficiency of recruitment, commentary on acceptability and new conceptual developments. These are discussed below:

Commentary on efficiency of recruitment

Unusually, no trials reported being unable to recruit to time and target(120).

Most articles commented on the efficiency of the design, enhanced recruitment, and the potential to embed in multiple RCTs. Young-Afat et al (121) reported that 88% of patients invited to join the UMBRELLA cohort consented to participate, most of whom (88%) gave broad consent for randomisation to future interventions. Coebergh van den Braak et al (122) proposed that the efficiency of this approach arises from physicians saving time by only explaining an intervention that they can *actually* offer to a patient. Viksveen et al (96) reported that the trial '*over-recruited*', and how the design enabled the acceptability of an intervention to be measured by intervention take-up in the intervention group.Viksveen et al(16) also argued that this approach has the potential to recruit more representative populations than stand-alone recruitment to an RCT and that the '*main benefits of using the cmRCT design to test an intervention for self-reported depression were full, fast and efficient recruitment; lower attrition rates than other depression trials; and a trial population broadly similar to the general population of patients self-reporting chronic moderate to severe depression*'(16).

For peer review only - http://bmjopen.bmj.com/site/about/guidelines.xhtml

Most trials (33/39) and all protocols stated that they had or would use an Intention to Treat (ITT) analysis as part of their statistical approach. Some also recommended using a complier average causal effect (CACE) analysis instead of per protocol analysis due to risk of bias(16, 96). In CACE analyses, outcomes in patients who take up the offer of treatment are compared to outcomes in the no offer group of patients who would have taken up the offer had they received it. Some authors used instrumental variables (IV) analysis, as this type of CACE-analysis takes into account patients' baseline characteristics(16, 37, 62, 68, 93) and commented that this analysis was appropriate where there is lower take-up in the intervention arm especially where the intervention is the offer of treatment.

One publication (Gal et al(50)), compared recruitment to a standalone RCT to recruitment from within a stage-consenting cohort. Recruitment from the stage-consenting cohort was significantly cheaper and faster and prevented contamination between groups '*but noncompliance due to refusal of the intervention was higher compared with conventional pragmatic exercise oncology RCTs, which may dilute the estimated intervention effect'*.

Commentary on acceptability of design

Several studies described Patient and Public Involvement (PPI) work around the trial design in the field of prostate cancer research (49) and kidney disease (45). An online survey conducted in collaboration with the charity Kidney Cancer UK to ascertain acceptability of the study's design and the willingness of patients to be recruited into the cohort found *'strong support and need from the kidney cancer patient community for the broad concepts of the study'*.

The first published evaluations of broad informed consent for randomisation within a TwiCs study were conducted in the Netherlands (52, 64). Cancer patients participating in ongoing TwiCs(52) and a separate study patients were surveyed between two to six years after cohort entry(64). The approach was found to be generally well accepted by patients with only 2% of patients in the trial usual care control group stating that they would feel negative about not being selected for interventions while their data are being used as a comparator. Both evaluations support the use of the TwiCs design with the staged-informed consent procedure.

New conceptual developments

Most cohorts using the TwiCs design approach were of clinical populations. Some publications described population cohorts e.g. England's 'Born in Bradford Better Start' (BIBBS) cohort (29),

For peer review only - http://bmjopen.bmj.com/site/about/guidelines.xhtml

the Australian 'Generation Victoria' population cohort (30). Some publicatons described '*multi-national*' cohorts - the Scleroderma Patient-centred Intervention Network (SPIN) cohort which involves 42 centres globally(84), the Emotional Competence in Young Adults (Ecoweb) PROMOTE platform to promote mental health in young adults (109) which uses a web based multi-national recruitment strategy/ The EPAD Project(80) is a large collaboration supporting a platform for the testing of multiple interventions. Solomon et al describe how *'As the EPAD project is multistaged, staged consent will be used as a decision-making model. Staged consent feeds relevant information—bit by bit, extended over time—to participants and study partners, and asks informed consent at every step when they need to make important decisions'(80).*

This review reports the characteristics of trials conducted using cohorts and observed how terminology and conceptual boundaries evolved over time in the methodological literature and the literature reporting research activity.

A diverse range of cohorts and trials using cohorts in a wide range of populations were identified. The TwiCs design approach is being utilised for a wide range of clinical conditions and interventions. A major clinical group is oncology (88, 107, 115, 123) in the Netherlands. Use of the TwiCs design is spreading with innovative work taking place in a range of locations and new collaborations are leading to the development of multi-national recruitment.

The term 'staged' approach to informed consent coined in 2016,(17) has replaced the earlier term 'patient-centred' in the majority of studies.

All the RCTs using the TwiCs design had usual care for at least one of the comparators - a prerequisite to using a staged approach, ie not to contact cohort participants further in the usual care group if they are not selected for trial interventions.

Either batch or sequential randomisation can be utilised as part of a TwiCs design depending on clinical condition and demands of study. However, the majority of protocols use sequential randomisation, where individuals are randomised as they become eligible for the experimental intervention (18).

The TwiCs approach has been cited as an example of an '*Optimization learning health care system*'(66) for encompassing strategies that contribute to a health care system in which *"the process of generating and applying the best evidence will be natural and seamless components of*

For peer review only - http://bmjopen.bmj.com/site/about/guidelines.xhtml

the process of care itself." Use of a staged informed consent process, routine data and individual randomisation at the point of diagnosis could all be considered examples of these strategies.

To-date the acceptability work around the TwiCs design has been mainly conducted in the field of oncology. Further work to assess the impact for different clinical groups is recommended. There is also a lack of direct comparison with standard RCTs.

This review confirms findings in the earlier scoping review(24) that there is a heterogeneity in the terminology and conceptual boundaries for the use of cohorts for RCTs. The term TwiCs is now used for the majority of studies but there is still some variation in terminology and use of the design. There are no current guidelines to support consistency of use.

CONCLUSION

This review identified increasing sophistication and maturity in RCTs conducted within cohorts in a wide range of conditions and research studies including 'mega-cohorts'(30). Our findings support the increased use of the TwiCs design to address some of the barriers to recruitment and retention for standard RCTs with usual care comparators. Further research into the acceptability, use and efficiency of staged approaches to informed consent, compared to standard approaches is required. We recommend the use of the term TwiCs to unify approaches and call for an international symposium to define terminology and develop guidelines for best practice for studies using the TwiCs approach.

4,199 words

Supplementary files

1. Electronic search strategies

Adapted from the published protocol(24). Searches were run in both MEDLINE and Cochrane Methodology Register simultaneously. As an example, in the cohort search, lines 1-11 are the MEDLINE search and lines 12-15 are tailored for the Cochrane Methodology Register. The final lines of each search isolate the records from each database, combine them so duplicate records can be removed, then isolate the remaining records so they can be downloaded and imported into Reference Manager using customized import filters.

Searches for RCTs embedded in Cohorts

1. (cohort adj5 (randomi#ed adj5 trial*)).ab,kf,ti.

2. (cohort adj5 RCT*).ab,kf,ti.

For peer review only - http://bmjopen.bmj.com/site/about/guidelines.xhtml

3. (cohort adj5 controlled trial*).ab,kf,ti.

4. (cmRCT or Cohort Multiple Randomised Controlled Trial*).ab,kf,ti.

5. or/1-4

6. cohort.af.

7. (embed* adj8 randomi#ed).ab,kf,ti.

8. (embed* adj8 RCT*).ab,kf,ti.

9. (embed* adj8 controlled trial*).ab,kf,ti.

10. or/7-9

11. 6 and 10

12. (pragmatic adj5 RCT*).ab,kf,ti.

13. (pragmatic adj5 randomi#ed).ab,kf,ti.

14. (pragmatic adj5 controlled trial*).ab,kf,ti.

15. or/12-14

16. 6 and 15

17. 5 or 11 or 16

18. (meta analy* or metaanaly* or metanaly* or systematic review*).af.

19. 17 not 18

20. limit 19 to yr="2007 - 2018"

21. ((Cohort* and (random* or RCT)) or cmRCT).ti,ab,kw.

22. limit 21 to yr="2007 - 2018"

23. 20 use medall

24. 22 use clcmr

25. 23 or 24

26. remove duplicates from 25

27. 26 use medall

28. 26 use clcmr

2. Inclusion/Exclusion criteria for studies

Adapted from protocol for a scoping review to support development of a CONSORT extension for randomised controlled trials using cohorts and routinely collected health data(24).

For peer review only

For peer review only - http://bmjopen.bmj.com/site/about/guidelines.xhtml

Exclude: The publication is NOT reporting trials conducted using cohorts, protocols for RCTs that will be conducted using cohorts, describing a cohort intended to be used or has been used to conduct RCTs or methodological papers describing the use of cohorts. If the study involves non-human subjects it will be excluded.

Include: The publication describes an RCT in which the cohort is used or will be used to identify trial participants. The publication describes a cohort used to identify trial participants

Include: The publication describes an RCT in which the cohort is used or will be used to ascertain health outcomes. The publication describes a cohort used to ascertain health outcomes.

Include: The methodological papers describe the use of cohorts for RCTs.

References

1. Barría RM. Introductory Chapter: The Contribution of Cohort Studies to Health Sciences. Cohort Studies in Health Sciences: InTech; 2018.
2. Relton C, Torgerson D, Cathain AO, Nicholl J. Rethinking pragmatic randomised controlled trials: introducing the "cohort multiple randomised controlled trial" design. BMJ. 2010;340:c1066.
3. Bower P, Brueton V, Gamble C, Treweek S, Smith CT, Young B, et al. Interventions to improve recruitment and retention in clinical trials: a survey and workshop to assess current practice and future priorities. Trials. 2014;15:399.
4. Watson JM, Torgerson DJ. Increasing recruitment to randomised trials: a review of randomised controlled trials. BMC Medical Research Methodology. 2006;6(1).
5. Campbell M, Snowdon C, Francis D, Elbourne D, Mcdonald A, Knight R, et al. Recruitment to randomised trials: strategies for trial enrolment and participation study. The STEPS study. Health Technology Assessment. 2007;11(48).
6. Treweek S, Lockhart P, Pitkethly M, Cook JA, Kjeldstrøm M, Johansen M, et al. Methods to improve recruitment to randomised controlled trials: Cochrane systematic review and meta-analysis. BMJ Open. 2013;3(2):e002360.
7. Sully BGO, Julious SA, Nicholl J. A reinvestigation of recruitment to randomised, controlled, multicenter trials: a review of trials funded by two UK funding agencies. Trials. 2013;14(1):166.
8. Mcdonald AM, Treweek S, Shakur H, Free C, Knight R, Speed C, et al. Using a business model approach and marketing techniques for recruitment to clinical trials. Trials. 2011;12(1):74.
9. Donovan JL, Paramasivan S, De Salis I, Toerien M. Clear obstacles and hidden challenges: understanding recruiter perspectives in six pragmatic randomised controlled trials. Trials. 2014;15(1):5.

For peer review only - http://bmjopen.bmj.com/site/about/guidelines.xhtml

10. Relton C, Thomas K, Nicholl J, Uher R. Review of an innovative approach to practical trials: the 'cohort multiple RCT' design. Trials. 2015;16(S2).
11. Relton C, Burbach M, Collett C, Flory J, Gerlich S, Holm S, et al. The ethics of 'Trials within Cohorts' (TwiCs): 2nd international symposium. Trials. 2017;18(S2).
12. Imran M, Mc Cord K, Mccall SJ, Kwakkenbos L, Sampson M, Fröbert O, et al. Reporting transparency and completeness in trials: Paper 3 – trials conducted using administrative databases do not adequately report elements related to use of databases. Journal of Clinical Epidemiology. 2022;141:187-97.
13. Mccall SJ, Imran M, Hemkens LG, Mc Cord K, Kwakkenbos L, Sampson M, et al. Reporting transparency and completeness in trials: Paper 4 - reporting of randomised controlled trials conducted using routinely collected electronic records – room for improvement. Journal of Clinical Epidemiology. 2022;141:198-209.
14. Mc Cord KA, Imran M, Rice DB, Mccall SJ, Kwakkenbos L, Sampson M, et al. Reporting transparency and completeness in Trials: Paper 2 - reporting of randomised trials using registries was often inadequate and hindered the interpretation of results. Journal of Clinical Epidemiology. 2021.
15. Mc Cord KA, Al-Shahi Salman R, Treweek S, Gardner H, Strech D, Whiteley W, et al. Routinely collected data for randomized trials: promises, barriers, and implications. Trials. 2018;19(1).
16. Viksveen P, Relton C, Nicholl J. Benefits and challenges of using the cohort multiple randomised controlled trial design for testing an intervention for depression. Trials [Electronic Resource]. 2017;18(1):308.
17. Young-Afat DA, Verkooijen HM, Gils CHV, er V, Burbach J, Elias SG, et al. Staged-informed consent in the cohort multiple randomized controlled trial design. Epidemiology. 2016;2016 Jan 06.
18. Couwenberg AM, Burbach JPM, May AM, Berbee M, Intven MPW, Verkooijen HM. The trials within cohorts design facilitated efficient patient enrollment and generalizability in oncology setting. Journal of Clinical Epidemiology. 2020;120:33-9.
19. Kwakkenbos L, Imran M, Mccall SJ, Mccord KA, Fröbert O, Hemkens LG, et al. CONSORT extension for the reporting of randomised controlled trials conducted using cohorts and routinely collected data (CONSORT-ROUTINE): checklist with explanation and elaboration. BMJ. 2021:n857.
20. Kwakkenbos L, Juszczak E, Hemkens LG, Sampson M, Frobert O, Relton C, et al. Protocol for the development of a CONSORT extension for RCTs using cohorts and routinely collected health data. Res Integr Peer Rev. 2018;3:9.
21. Tricco AC, Lillie E, Zarin W, O'Brien KK, Colquhoun H, Levac D, et al. PRISMA Extension for Scoping Reviews (PRISMA-ScR): Checklist and Explanation. Annals of Internal Medicine. 2018;169(7):467.
22. Nickolls B, Relton C, Eldridge S, Zwarenstein M, McCall S, Hemkens L, et al. Protocol for a scoping review of randomised controlled trials conducted using cohorts. Open Science Framework. 2021.
23. Colquhoun HL, Levac D, O'Brien KK, Straus S, Tricco AC, Perrier L, et al. Scoping reviews: time for clarity in definition, methods, and reporting. Journal of Clinical Epidemiology. 2014;67(12):1291-4.
24. Kwakkenbos L, Imran M, McCord KA, Sampson M, Frobert O, Gale C, et al. Protocol for a scoping review to support development of a CONSORT extension for randomised controlled trials using cohorts and routinely collected health data. BMJ Open. 2018;8(8):e025266.
25. Mcgowan J, Sampson M, Salzwedel DM, Cogo E, Foerster V, Lefebvre C. PRESS Peer Review of Electronic Search Strategies: 2015 Guideline Statement. Journal of Clinical Epidemiology. 2016;75:40-6.
26. Khangura S, Konnyu K, Cushman R, Grimshaw J, Moher D. Evidence summaries: the evolution of a rapid review approach. Systematic Reviews. 2012;1(1):10.
27. ISRCTN. Registry Definitions: Biomed Central Ltd 2020 [cited 2021 January 12th]. Available from: http://www.isrctn.com/page/definitions.
28. Martineau PA. COVIDENCE UK - National longitudinal Study of COVID-19. SMD COVID Research Seminar Series. UK: Queen Mary University London; 2020.
29. Dickerson J, Bird PK, McEachan RR, Pickett KE, Waiblinger D, Uphoff E, et al. Born in Bradford's Better Start: an experimental birth cohort study to evaluate the impact of early life interventions. BMC Public Health. 2016;15:711.

For peer review only - http://bmjopen.bmj.com/site/about/guidelines.xhtml

30. Wake M, Hu YJ, Warren H, Danchin M, Fahey M, Orsini F, et al. Integrating trials into a whole-population cohort of children and parents: statement of intent (trials) for the Generation Victoria (GenV) cohort. BMC Medical Research Methodology. 2020;20(1).
31. Snider C, Jiang D, Logsetty S, Strome T, Klassen T. Wraparound care for youth injured by violence: study protocol for a pilot randomised control trial. BMJ Open. 2015;5(5):e008088-e.
32. Finucane FM, Sharp SJ, Purslow LR, Horton K, Horton J, Savage DB, et al. The effects of aerobic exercise on metabolic risk, insulin sensitivity and intrahepatic lipid in healthy older people from the Hertfordshire Cohort Study: a randomised controlled trial. Diabetologia. 2010;53(4):624-31.
33. Fibert P, Relton C, Peasgood T, Daley D. Protocol for the STAR (Sheffield Treatments for ADHD) project: an internal pilot study assessing the feasibility of the Trials within Cohorts (TwiCs) design to test the effectiveness of interventions for children with ADHD. Pilot Feasibility Stud. 2018;4:61.
34. Hafkamp-De Groen E, Mohangoo AD, De Jongste JC, Van Der Wouden JC, Moll HA, Jaddoe VW, et al. Early detection and counselling intervention of asthma symptoms in preschool children: study design of a cluster randomised controlled trial. BMC Public Health. 2010;10(1):555.
35. Relton C, Bissell P, Smith C, Blackburn J, Cooper CL, Nicholl J, et al. South Yorkshire Cohort: a 'cohort trials facility' study of health and weight - protocol for the recruitment phase. BMC Public Health. 2011;11:640.
36. Finer S, Martin HC, Khan A, Hunt KA, Maclaughlin B, Ahmed Z, et al. Cohort Profile: East London Genes & Health (ELGH), a community-based population genomics and health study in British Bangladeshi and British Pakistani people. International Journal of Epidemiology. 2020;49(1):20-1i.
37. Panagioti M, Reeves D, Meacock R, Parkinson B, Lovell K, Hann M, et al. Is telephone health coaching a useful population health strategy for supporting older people with multimorbidity? An evaluation of reach, effectiveness and cost-effectiveness using a 'trial within a cohort'. BMC Medicine. 2018;16(1).
38. Fibert P, Peasgood T, Relton C. Rethinking ADHD intervention trials: feasibility testing of two treatments and a methodology. Eur J Pediatr. 2019.
39. van Wijk RM, van Vlimmeren LA, Groothuis-Oudshoorn CG, Van der Ploeg CP, Ijzerman MJ, Boere-Boonekamp MM. Helmet therapy in infants with positional skull deformation: randomised controlled trial. BMJ. 2014;348:g2741.
40. Grady KFO, Grimwood K, Toombs M, Sloots TP, Otim M, Whiley D, et al. Effectiveness of a cough management algorithm at the transitional phase from acute to chronic cough in Australian children aged <15 years: protocol for a randomised controlled trial. BMJ Open. 2017;7(3):e013796.
41. Jordan S, Gabe-Walters ME, Watkins A, Humphreys I, Newson L, Snelgrove S, et al. Nurse-Led Medicines' Monitoring for Patients with Dementia in Care Homes: A Pragmatic Cohort Stepped Wedge Cluster Randomised Trial. PLoS One. 2015;10(10):e0140203.
42. Beekman E, Mesters I, Hendriks EJ, Muris JW, Wesseling G, Evers SM, et al. Exacerbations in patients with chronic obstructive pulmonary disease receiving physical therapy: a cohort-nested randomised controlled trial. BMC Pulmonary Medicine. 2014;14:71.
43. Sihvonen R, Paavola M, Malmivaara A, Jarvinen TL. Finnish Degenerative Meniscal Lesion Study (FIDELITY): a protocol for a randomised, placebo surgery controlled trial on the efficacy of arthroscopic partial meniscectomy for patients with degenerative meniscus injury with a novel 'RCT within-a-cohort' study design. BMJ Open. 2013;3(3).
44. Tofte N, Lindhardt M, Adamova K, Bakker SJL, Beige J, Beulens JWJ, et al. Early detection of diabetic kidney disease by urinary proteomics and subsequent intervention with spironolactone to delay progression (PRIORITY): a prospective observational study and embedded randomised placebo-controlled trial. The Lancet Diabetes & Endocrinology. 2020;8(4):301-12.
45. Freedman DM, Kuncl RW, Weinstein SJ, Malila N, Virtamo J, Albanes D. Vitamin E serum levels and controlled supplementation and risk of amyotrophic lateral sclerosis. Amyotrophic Lateral sclerosis & Frontotemporal Degeneration. 2013;14(4):246.
46. Kazemier BM, Koningstein FN, Schneeberger C, Ott A, Bossuyt PM, de Miranda E, et al. Maternal and neonatal consequences of treated and untreated asymptomatic bacteriuria in pregnancy: a

For peer review only - http://bmjopen.bmj.com/site/about/guidelines.xhtml

prospective cohort study with an embedded randomised controlled trial. Lancet Infect Dis. 2015;15(11):1324-33.
47. Sheeran L, Deursen Rv, Caterson B, Sparkes V. Classification-guided versus generalized postural intervention in subgroups of nonspecific chronic low back pain: a pragmatic randomized controlled study. Spine. 2013;38(19):1613.
48. Renteln-Kruse Wv, Dapp U, Anders J, Profener F, Schmidt S, Deneke C, et al. The LUCAS* consortium: objectives of interdisciplinary research on selected aspects of ageing and health care for older people in an urban community. Zeitschrift fur Gerontologie und Geriatrie. 2011;44(4):250.
49. Ahmed HU, Berge V, Bottomley D, Cross W, Heer R, Kaplan R, et al. Can we deliver randomized trials of focal therapy in prostate cancer?. [Review][Erratum appears in Nat Rev Clin Oncol. 2017 Sep 12;:; PMID: 28895571]. Nature Reviews Clinical Oncology. 2014;11(8):482.
50. Gal R, Monninkhof EM, Groenwold RHH, Van Gils CH, Van Den Bongard DHJG, Peeters PHM, et al. The effects of exercise on the quality of life of patients with breast cancer (the UMBRELLA Fit study): study protocol for a randomized controlled trial. Trials. 2017;18(1).
51. Heaven A, Brown L, Foster M, Clegg A. Keeping it credible in cohort multiple Randomised Controlled Trials: the Community Ageing Research 75+ (CARE 75+) study model of patient and public involvement and engagement. Research Involvement & Engagement. 2016;2:30.
52. Young-Afat DA, Gal R, Gerlich S, Burbach JPM, Van Der Velden JM, Van Den Bongard DHJG, et al. Oncology patients were found to understand and accept the Trials within Cohorts design. Journal of Clinical Epidemiology. 2021;130:135-42.
53. Zakrzewska JM, Relton C. Future Directions for Surgical Trial Designs in Trigeminal Neuralgia. [Review]. Neurosurgery Clinics of North America. 2016;27(3):353.
54. Richards DA, Ross S, Robens S, Borglin G. The DiReCT study - improving recruitment into clinical trials: a mixed methods study investigating the ethical acceptability, feasibility and recruitment yield of the cohort multiple randomised controlled trials design. Trials [Electronic Resource]. 2014;15:398.
55. Candlish J, Pate A, Sperrin M, Staa Tv, Work Package G. Evaluation of biases present in the cohort multiple randomised controlled trial design: a simulation study.[Erratum appears in BMC Med Res Methodol. 2017 Mar 22;17 (1):47; PMID: 28330450]. BMC Medical Research Methodology. 2017;17(1):17.
56. Clegg A, Relton C, Young J, Witham M. Improving recruitment of older people to clinical trials: use of the cohort multiple randomised controlled trial design. Age Ageing. 2015;44(4):547-50.
57. Flory JH, Mushlin AI, Goodman ZI. Proposals to Conduct Randomized Controlled Trials Without Informed Consent: a Narrative Review. [Review]. Journal of General Internal Medicine. 2016;31(12):1511.
58. Kim SY, Flory J, Relton C. Ethics and practice of Trials within Cohorts: An emerging pragmatic trial design. Clin Trials. 2018;15(1):9-16.
59. Lambin P, Zindler J, Vanneste B, e v, Jacobs M, Eekers D, et al. Modern clinical research: How rapid learning health care and cohort multiple randomised clinical trials complement traditional evidence based medicine. Acta Oncologica. 2015;54(9):1289.
60. Pate A, Candlish J, Sperrin M, Staa TPV, Work Package G. Cohort Multiple Randomised Controlled Trials (cmRCT) design: efficient but biased? A simulation study to evaluate the feasibility of the Cluster cmRCT design. BMC Medical Research Methodology. 2016;16(1):109.
61. Reeves D, Howells K, Sidaway M, Blakemore A, Hann M, Panagioti M, et al. The cohort multiple randomized controlled trial design was found to be highly susceptible to low statistical power and internal validity biases. Journal of Clinical Epidemiology. 2018;95:111.
62. van der Velden JM, Verkooijen HM, Young-Afat DA, Burbach JP, van Vulpen M, Relton C, et al. The cohort multiple randomized controlled trial design: a valid and efficient alternative to pragmatic trials? Int J Epidemiol. 2017;46(1):96-102.
63. Verkooijen HM, Roes K, van Gils CH. [Cohort multiple randomized controlled trial: a solution for the evaluation of multiple interventions]. Ned Tijdschr Geneeskd. 2013;157(17):A5762.

For peer review only - http://bmjopen.bmj.com/site/about/guidelines.xhtml

64. Verweij ME, Gal R, Burbach JPM, Young-Afat DA, Van Der Velden JM, Van Der Graaf R, et al. Most patients reported positively or neutrally of having served as controls in the trials within cohorts design. Journal of Clinical Epidemiology. 2022;148:39-47.
65. Vickers AJ, Young-Afat DA, Ehdaie B, Kim SY. Just-in-time consent: The ethical case for an alternative to traditional informed consent in randomized trials comparing an experimental intervention with usual care. Clin Trials. 2018;15(1):3-8.
66. Wouters. Learning health care systems: Highly needed but challenging. Wiley Online Library. 2020.
67. Arundel C, Jefferson L, Bailey M, Cockayne S, Hicks K, Loughrey L, et al. A randomized, embedded trial of pre-notification of trial participation did not increase recruitment rates to a falls prevention trial. Journal of Evaluation in Clinical Practice. 2017;23(1):73.
68. Cockayne S, Adamson J, Clarke A, Corbacho B, Fairhurst C, Green L, et al. Cohort Randomised Controlled Trial of a Multifaceted Podiatry Intervention for the Prevention of Falls in Older People (The REFORM Trial). PLoS ONE [Electronic Resource]. 2017;12(1):e0168712.
69. Cotterill S, Howells K, Rhodes S, Bower P. The effect of using social pressure in cover letters to improve retention in a longitudinal health study: an embedded randomised controlled retention trial. Trials. 2017;18(1).
70. Boyd A, Tilling K, Cornish R, Davies A, Humphries K, Macleod J. Professionally designed information materials and telephone reminders improved consent response rates: evidence from an RCT nested within a cohort study. Journal of Clinical Epidemiology. 2015;68(8):877.
71. Goodwin M, Walsh T, Whittaker W, Emsley R, Sutton M, Tickle M, et al. Increasing questionnaire response: evidence from a nested RCT within a longitudinal birth cohort study. BMC Medical Research Methodology. 2020;20(1).
72. Knapp P, Raynor DK, Silcock J, Parkinson B. Can user testing of a clinical trial patient information sheet make it fit-for-purpose? - a randomized controlled trial. BMC Medicine. 2011;9(1):89.
73. Ni MY, Li TK, Hui RWH, McDowell I, Leung GM. Requesting a unique personal identifier or providing a souvenir incentive did not affect overall consent to health record linkage: evidence from an RCT nested within a cohort. Journal of Clinical Epidemiology. 2017;84:142.
74. Loban A, Mandefield L, Hind D, Bradburn M. A randomized trial found online questionnaires supplemented by postal reminders generated a cost-effective and generalizable sample but don't forget the reminders. Journal of Clinical Epidemiology. 2017;92:116-25.
75. Maruani A, Boutron I, Baron G, Ravaud P. Impact of sending email reminders of the legal requirement for posting results on ClinicalTrials.gov: cohort embedded pragmatic randomized controlled trial. BMJ. 2014;349:g5579.
76. Collins K, Reed M, Lifford K, Burton M, Edwards A, Ring A, et al. Bridging the age gap in breast cancer: evaluation of decision support interventions for older women with operable breast cancer: protocol for a cluster randomised controlled trial. BMJ Open. 2017;7(7):e015133.
77. Burchell AN, Allen VG, Grewal R, Macpherson PA, Rachlis A, Walmsley S, et al. Enhanced syphilis screening among HIV-positive men (ESSAHM): a study protocol for a clinic-randomized trial with stepped wedge design. Implementation Science. 2015;11(1).
78. Hestbaek L, Andersen ST, Skovgaard T, Olesen LG, Elmose M, Bleses D, et al. Influence of motor skills training on children's development evaluated in the Motor skills in PreSchool (MiPS) study-DK: study protocol for a randomized controlled trial, nested in a cohort study. Trials [Electronic Resource]. 2017;18(1):400.
79. Shields N, Willis C, Imms C, Prendergast LA, Watts JJ, Van Dorsselaer B, et al. FitSkills: protocol for a stepped wedge cluster randomised trial of a community-based exercise programme to increase participation among young people with disability. BMJ Open. 2020;10(7):e037153.
80. Solomon A, Kivipelto M, Molinuevo JL, Tom B, Ritchie CW. European Prevention of Alzheimer's Dementia Longitudinal Cohort Study (EPAD LCS): study protocol. BMJ Open. 2018;8(12):e021017.

For peer review only - http://bmjopen.bmj.com/site/about/guidelines.xhtml

81. er V, Verkooijen HM, Young-Afat DA, Burbach JP, Vulpen MV, Relton C, et al. The cohort multiple randomized controlled trial design: a valid and efficient alternative to pragmatic trials? International Journal of Epidemiology. 2017;46(1):96.
82. Mitchell N, Hewitt C, Adamson J, Parrott S, Torgerson D, Ekers D, et al. A randomised evaluation of CollAborative care and active surveillance for Screen-Positive EldeRs with sub-threshold depression (CASPER): study protocol for a randomized controlled trial. Trials [Electronic Resource]. 2011;12:225.
83. Peckham EJ, Relton C, Raw J, Walters C, Thomas K, Smith C. A protocol for a trial of homeopathic treatment for irritable bowel syndrome. BMC Complementary & Alternative Medicine. 2012;12:212.
84. Kwakkenbos L, Jewett LR, Baron M, Bartlett SJ, Furst D, Gottesman K, et al. The Scleroderma Patient-centered Intervention Network (SPIN) Cohort: protocol for a cohort multiple randomised controlled trial (cmRCT) design to support trials of psychosocial and rehabilitation interventions in a rare disease context. BMJ Open. 2013;3(8).
85. Uher R, Cumby J, MacKenzie LE, Morash-Conway J, Glover JM, Aylott A, et al. A familial risk enriched cohort as a platform for testing early interventions to prevent severe mental illness. BMC Psychiatry. 2014;14:344.
86. Viksveen P, Relton C. Depression treated by homeopaths: a study protocol for a pragmatic cohort multiple randomised controlled trial. Homeopathy: the Journal of the Faculty of Homeopathy. 2014;103(2):147.
87. Burbach JP, Verkooijen HM, Intven M, Kleijnen JP, Bosman ME, Raaymakers BW, et al. RandomizEd controlled trial for pre-operAtive dose-escaLation BOOST in locally advanced rectal cancer (RECTAL BOOST study): study protocol for a randomized controlled trial. Trials [Electronic Resource]. 2015;16:58.
88. Couwenberg AM, Burbach MJ, Smits AB, Van Vulpen M, Van Grevenstein WM, Noordzij PG, et al. The impact of retractor SPONGE-assisted laparoscopic surgery on duration of hospital stay and postoperative complications in patients with colorectal cancer (SPONGE trial): study protocol for a randomized controlled trial. Trials. 2016;17(1):132.
89. van der Velden JM, Verkooijen HM, Seravalli E, Hes J, Gerlich AS, Kasperts N, et al. Comparing conVEntional RadioTherapy with stereotactIC body radiotherapy in patients with spinAL metastases: study protocol for an randomized controlled trial following the cohort multiple randomized controlled trial design. BMC Cancer. 2016;16(1):909.
90. Lee H, Wiggers J, Kamper SJ, Williams A, Brien KMO, Hodder RK, et al. Mechanism evaluation of a lifestyle intervention for patients with musculoskeletal pain who are overweight or obese: protocol for a causal mediation analysis. BMJ Open. 2017;7(6):e014652.
91. Brunahl CA, Klotz SGR, Dybowski C, Riegel B, Gregorzik S, Tripp DA, et al. Combined Cognitive-Behavioural and Physiotherapeutic Therapy for Patients with Chronic Pelvic Pain Syndrome (COMBI-CPPS): study protocol for a controlled feasibility trial. Trials [Electronic Resource]. 2018;19(1):20.
92. Anderson BM, Dutton M, Day E, Jackson TA, Ferro CJ, Sharif A. Frailty Intervention Trial iN End-Stage patientS on haemodialysis (FITNESS): study protocol for a randomised controlled trial. Trials. 2018;19(1).
93. Relton C, Cathain AO, Nicholl J. A pilot 'cohort multiple randomised controlled trial' of treatment by a homeopath for women with menopausal hot flushes. Contemporary Clinical Trials. 2012;33(5):853.
94. Dascanio V, Birks Y, Clark L, Fairhurst C, MacPherson H, Torgerson DJ. Randomized cohort trial was shown to be feasible for evaluating treatments in low back pain. Journal of Clinical Epidemiology. 2014;67(8):940.
95. Cockayne S, Rodgers S, Green L, Fairhurst C, Adamson J, Scantlebury A, et al. Clinical effectiveness and cost-effectiveness of a multifaceted podiatry intervention for falls prevention in older people: a multicentre cohort randomised controlled trial (the REducing Falls with ORthoses and a Multifaceted podiatry intervention trial). Health Technology Assessment (Winchester, England). 2017;21(24):1.

For peer review only - http://bmjopen.bmj.com/site/about/guidelines.xhtml

96. Viksveen P, Relton C, Nicholl J. Depressed patients treated by homeopaths: a randomised controlled trial using the "cohort multiple randomised controlled trial" (cmRCT) design. Trials. 2017;18(1):299.
97. Brien KMO, Wiggers J, Williams A, Campbell E, Hodder RK, Wolfenden L, et al. Telephone-based weight loss support for patients with knee osteoarthritis: a pragmatic randomised controlled trial. Osteoarthritis & Cartilage. 2018;26(4):485.
98. Skaaby T, Jorgensen T, Linneberg A. A randomized general population study of the effects of repeated health checks on incident diabetes. Endocrine. 2018;60(1):122-8.
99. Williams A, Wiggers J, Brien KMO, Wolfenden L, Yoong SL, Hodder RK, et al. The effectiveness of a healthy lifestyle intervention, for chronic low back pain: a randomised controlled trial. Pain. 2018;2018 Mar 08.
100. Bibby AC, Torgerson DJ, Leach S, Lewis-White H, Maskell NA. Commentary: considerations for using the 'Trials within Cohorts' design in a clinical trial of an investigational medicinal product. Trials [Electronic Resource]. 2018;19(1):18.
101. Gal R, Monninkhof EM, Van Gils CH, Groenwold RHH, Elias SG, Van Den Bongard DHJG, et al. Effects of exercise in breast cancer patients: implications of the trials within cohorts (TwiCs) design in the UMBRELLA Fit trial. Breast Cancer Research and Treatment. 2021;190(1):89-101.
102. Vickers AJ, Vertosick EA, Carlsson SV, Ehdaie B, Kim SYH. Patient accrual and understanding of informed consent in a two-stage consent design. Clin Trials. 2021;18(3):377-82.
103. Mayo NE, Brouillette MJ, Fellows LK, Positive Brain Health Now I. Understanding and optimizing brain health in HIV now: protocol for a longitudinal cohort study with multiple randomized controlled trials. BMC Neurology. 2016;16:8.
104. Drew CJG, Quinn L, Hamana K, Williams-Thomas R, Marsh L, Dimitropoulou P, et al. Physical Activity and Exercise Outcomes in Huntington Disease (PACE-HD): Protocol for a 12-Month Trial Within Cohort Evaluation of a Physical Activity Intervention in People With Huntington Disease. Physical Therapy. 2019;99(9):1201-10.
105. Heaven A, Brown L, Young J, Teale E, Hawkins R, Spilsbury K, et al. Community ageing research 75+ study (CARE75+): an experimental ageing and frailty research cohort. BMJ Open. 2019;9(3):e026744.
106. Wylie H, Cahill F, Santaolalla A, Moss CL, Enting D, Amery S, et al. Graham Roberts Study protocol: first 'trials within cohort study' for bladder cancer. BMJ Open. 2019;9(9):e029468.
107. Batenburg MCT, Van Den Bongard HJGD, Kleynen CE, Maarse W, Witkamp A, Ernst M, et al. Assessing the effect of hyperbaric oxygen therapy in breast cancer patients with late radiation toxicity (HONEY trial): a trial protocol using a trial within a cohort design. Trials. 2020;21(1).
108. Lee RZY, Yu J, Rawtaer I, Allen PF, Bao Z, Feng L, et al. CHI study: protocol for an observational cohort study on ageing and mental health in community-dwelling older adults. BMJ Open. 2020;10(5):e035003.
109. Newbold A, Warren FC, Taylor RS, Hulme C, Burnett S, Aas B, et al. Promotion of mental health in young adults via mobile phone app: study protocol of the ECoWeB (emotional competence for well-being in Young adults) cohort multiple randomised trials. BMC Psychiatry. 2020;20(1).
110. Schraa SJ, Van Rooijen KL, Van Der Kruijssen DEW, Rubio Alarcón C, Phallen J, Sausen M, et al. Circulating tumor DNA guided adjuvant chemotherapy in stage II colon cancer (MEDOCC-CrEATE): study protocol for a trial within a cohort study. BMC Cancer. 2020;20(1).
111. Brown L, Heaven A, Quinn C, Goodwin V, Chew-Graham C, Mahmood F, et al. Community Ageing Research 75+ (CARE75+) REMOTE study: a remote model of recruitment and assessment of the health, well-being and social circumstances of older people. BMJ Open. 2021;11(11):e048524.
112. Richters A, Meijer RP, Mehra N, Boormans JL, Van Der Heijden AG, Van Der Heijden MS, et al. Prospective bladder cancer infrastructure for experimental and observational research on bladder cancer: study protocol for the 'trials within cohorts' study ProBCI. BMJ Open. 2021;11(5):e047256.

For peer review only - http://bmjopen.bmj.com/site/about/guidelines.xhtml

113. Holt H, Relton C, Talaei M, Symons J, Davies MR, Jolliffe DA, et al. Cohort Profile: Longitudinal population-based study of COVID-19 in UK adults (COVIDENCE UK). International Journal of Epidemiology. 2022.
114. Witlox L, Hiensch AE, Velthuis MJ, Steins Bisschop CN, Los M, Erdkamp FLG, et al. Four-year effects of exercise on fatigue and physical activity in patients with cancer. BMC Medicine. 2018;16(1).
115. Couwenberg AM, Burbach JPM, Berbee M, Lacle MM, Arensman R, Raicu MG, et al. Efficacy of Dose-Escalated Chemoradiation on Complete Tumor Response in Patients with Locally Advanced Rectal Cancer (RECTAL-BOOST): A Phase 2 Randomized Controlled Trial. International Journal of Radiation Oncology*Biology*Physics. 2020;108(4):1008-18.
116. Pielkenrood BJ, Van Der Velden JM, Van Der Linden YM, Bartels MMT, Kasperts N, Verhoeff JJC, et al. Pain Response After Stereotactic Body Radiation Therapy Versus Conventional Radiation Therapy in Patients With Bone Metastases—A Phase 2 Randomized Controlled Trial Within a Prospective Cohort. International Journal of Radiation Oncology*Biology*Physics. 2020.
117. Grimmer K, Luker J, Beaton K, Kumar S, Crockett A, Price K. TRialing individualized interventions to prevent functional decline in at-risk older adults (TRIIFL): study protocol for a randomized controlled trial nested in a longitudinal observational study. Trials [Electronic Resource]. 2013;14:266.
118. Merat S, Poustchi H, Hemming K, Jafari E, Radmard AR, Nateghi A, et al. PolyPill for Prevention of Cardiovascular Disease in an Urban Iranian Population with Special Focus on Nonalcoholic Steatohepatitis: A Pragmatic Randomized Controlled Trial within a Cohort (PolyIran - Liver) - Study Protocol. Archives of Iranian Medicine. 2015;18(8):515.
119. Neves JB, Cullen D, Grant L, Walkden M, Bandula S, Patki P, et al. Protocol for a feasibility study of a cohort embedded randomised controlled trial comparingNEphronSparingTreatment (NEST) for small renal masses. BMJ Open. 2019;9(6):e030965.
120. Desai M. Recruitment and retention of participants in clinical studies: Critical issues and challenges. Perspect Clin Res. 2020;11(2):51-3.
121. Young-Afat DA, van Gils CH, van den Bongard H, Verkooijen HM, Group US. The Utrecht cohort for Multiple BREast cancer intervention studies and Long-term evaLuAtion (UMBRELLA): objectives, design, and baseline results. Breast Cancer Res Treat. 2017;164(2):445-50.
122. Coebergh van den Braak RRJ, Rijssen LBv, Kleef JJv, Vink GR, Berbee M, Henegouwen MIvB, et al. Nationwide comprehensive gastro-intestinal cancer cohorts: the 3P initiative. Acta Oncologica. 2018;57(2):195.
123. er V, Verkooijen HM, Seravalli E, Hes J, Gerlich AS, Kasperts N, et al. Comparing conVEntional RadioTherapy with stereotactIC body radiotherapy in patients with spinAL metastases: study protocol for an randomized controlled trial following the cohort multiple randomized controlled trial design. BMC Cancer. 2016;16(1):909.

For peer review only - http://bmjopen.bmj.com/site/about/guidelines.xhtml

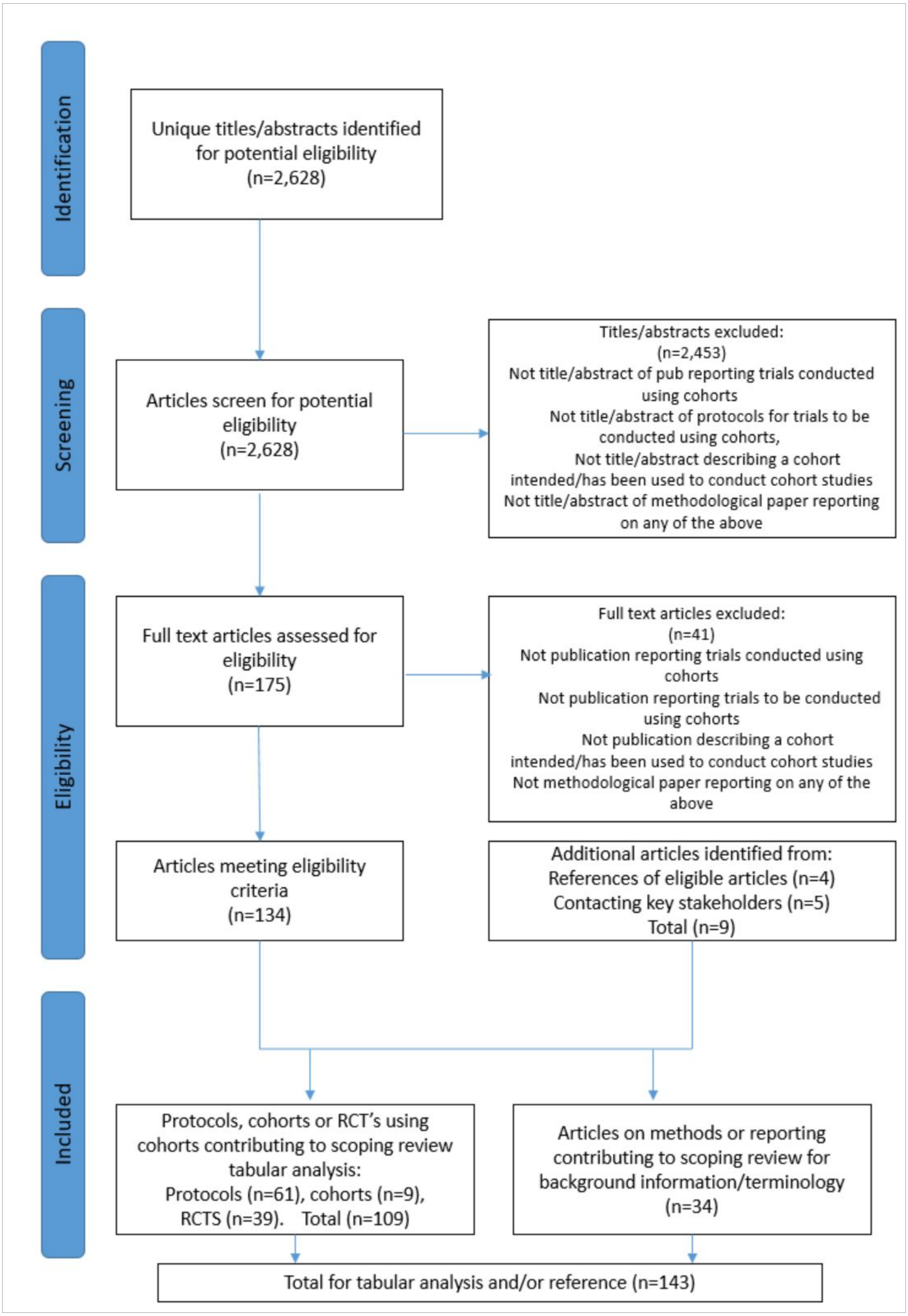
Identification
Unique titles/abstracts identified for potential eligibility (n=2,628)
Screening
Articles screen for potential eligibility (n=2,628)
Titles/abstracts excluded: (n=2,453)
Not title/abstract of pub reporting trials conducted using cohorts
Not title/abstract of protocols for trials to be conducted using cohorts,
Not title/abstract describing a cohort intended/has been used to conduct cohort studies
Not title/abstract of methodological paper reporting on any of the above
Eligibility
Full text articles assessed for eligibility (n=175)
Full text articles excluded: (n=41)
Not publication reporting trials conducted using cohorts
Not publication reporting trials to be conducted using cohorts
Not publication describing a cohort intended/has been used to conduct cohort studies
Not methodological paper reporting on any of the above
Articles meeting eligibility criteria (n=134)
Additional articles identified from:
References of eligible articles (n=4)
Contacting key stakeholders (n=5)
Total (n=9)
Included
Protocols, cohorts or RCT's using cohorts contributing to scoping review tabular analysis:
Protocols (n=61), cohorts (n=9), RCTS (n=39). Total (n=109)
Articles on methods or reporting contributing to scoping review for background information/terminology (n=34)
Total for tabular analysis and/or reference (n=143)

Preferred Reporting Items for Systematic reviews and Meta-Analyses extension for Scoping Reviews (PRISMA-ScR) Checklist

SECTION	ITEM	PRISMA-ScR CHECKLIST ITEM	REPORTED ON PAGE #
**TITLE**			
Title	1	Identify the report as a scoping review.	
**ABSTRACT**			
Structured summary	2	Provide a structured summary that includes (as applicable): background, objectives, eligibility criteria, sources of evidence, charting methods, results, and conclusions that relate to the review questions and objectives.	
**INTRODUCTION**			
Rationale	3	Describe the rationale for the review in the context of what is already known. Explain why the review questions/objectives lend themselves to a scoping review approach.	
Objectives	4	Provide an explicit statement of the questions and objectives being addressed with reference to their key elements (e.g., population or participants, concepts, and context) or other relevant key elements used to conceptualize the review questions and/or objectives.	
**METHODS**			
Protocol and registration	5	Indicate whether a review protocol exists; state if and where it can be accessed (e.g., a Web address); and if available, provide registration information, including the registration number.	
Eligibility criteria	6	Specify characteristics of the sources of evidence used as eligibility criteria (e.g., years considered, language, and publication status), and provide a rationale.	
Information sources*	7	Describe all information sources in the search (e.g., databases with dates of coverage and contact with authors to identify additional sources), as well as the date the most recent search was executed.	
Search	8	Present the full electronic search strategy for at least 1 database, including any limits used, such that it could be repeated.	
Selection of sources of evidence†	9	State the process for selecting sources of evidence (i.e., screening and eligibility) included in the scoping review.	
Data charting process‡	10	Describe the methods of charting data from the included sources of evidence (e.g., calibrated forms or forms that have been tested by the team before their use, and whether data charting was done independently or in duplicate) and any processes for obtaining and confirming data from investigators.	
Data items	11	List and define all variables for which data were sought and any assumptions and simplifications made.	
Critical appraisal of individual sources of evidence§	12	If done, provide a rationale for conducting a critical appraisal of included sources of evidence; describe the methods used and how this information was used in any data synthesis (if appropriate).	
Synthesis of results	13	Describe the methods of handling and summarizing the data that were charted.	

St. Michael's
Inspired Care.
Inspiring Science.

For peer review only - http://bmjopen.bmj.com/site/about/guidelines.xhtml

SECTION	ITEM	PRISMA-ScR CHECKLIST ITEM	REPORTED ON PAGE #
**RESULTS**			
Selection of sources of evidence	14	Give numbers of sources of evidence screened, assessed for eligibility, and included in the review, with reasons for exclusions at each stage, ideally using a flow diagram.	
Characteristics of sources of evidence	15	For each source of evidence, present characteristics for which data were charted and provide the citations.	
Critical appraisal within sources of evidence	16	If done, present data on critical appraisal of included sources of evidence (see item 12).	
Results of individual sources of evidence	17	For each included source of evidence, present the relevant data that were charted that relate to the review questions and objectives.	
Synthesis of results	18	Summarize and/or present the charting results as they relate to the review questions and objectives.	
**DISCUSSION**			
Summary of evidence	19	Summarize the main results (including an overview of concepts, themes, and types of evidence available), link to the review questions and objectives, and consider the relevance to key groups.	
Limitations	20	Discuss the limitations of the scoping review process.	
Conclusions	21	Provide a general interpretation of the results with respect to the review questions and objectives, as well as potential implications and/or next steps.	
**FUNDING**			
Funding	22	Describe sources of funding for the included sources of evidence, as well as sources of funding for the scoping review. Describe the role of the funders of the scoping review.	

JBI = Joanna Briggs Institute; PRISMA-ScR = Preferred Reporting Items for Systematic reviews and Meta-Analyses extension for Scoping Reviews.
* Where *sources of evidence* (see second footnote) are compiled from, such as bibliographic databases, social media platforms, and Web sites.
† A more inclusive/heterogeneous term used to account for the different types of evidence or data sources (e.g., quantitative and/or qualitative research, expert opinion, and policy documents) that may be eligible in a scoping review as opposed to only studies. This is not to be confused with *information sources* (see first footnote).
‡ The frameworks by Arksey and O'Malley (6) and Levac and colleagues (7) and the JBI guidance (4, 5) refer to the process of data extraction in a scoping review as data charting.
§ The process of systematically examining research evidence to assess its validity, results, and relevance before using it to inform a decision. This term is used for items 12 and 19 instead of "risk of bias" (which is more applicable to systematic reviews of interventions) to include and acknowledge the various sources of evidence that may be used in a scoping review (e.g., quantitative and/or qualitative research, expert opinion, and policy document).

From: Tricco AC, Lillie E, Zarin W, O'Brien KK, Colquhoun H, Levac D, et al. PRISMA Extension for Scoping Reviews (PRISMAScR): Checklist and Explanation. Ann Intern Med. 2018;169:467–473. doi: 10.7326/M18-0850.

For peer review only

BMJ Open

Randomised trials conducted using cohorts: A scoping review

Journal:	*BMJ Open*
Manuscript ID	bmjopen-2023-075601.R1
Article Type:	Original research
Date Submitted by the Author:	23-Nov-2023

Complete List of Authors:	Nickolls, Beverley; Queen Mary University of London, Centre for Evaluation and Methods, Wolfson Institute of Population Health<br>Relton, Clare ; Queen Mary University of London, Centre for Evaluation and Methods, Wolfson Institute of Population Health<br>Hemkens, Lars; University Hospital Basel, Research Center for Clinical Neuroimmunology and Neuroscience Basel (RC2NB); University of Basel, Department of Clinical Research<br>Zwarenstein, Merrick; Western University, Department of Family Medicine; Institute for Clinical Evaluative Sciences<br>Eldridge, Sandra; Queen Mary University of London, Centre for Evaluation and Methods, Wolfson Institute of Population Health<br>McCall, Stephen; University of Oxford, National Perinatal Epidemiology Unit, Clinical Trials Unit, Nuffield Department of Population Health; American University of Beirut, Center for Research on Population and Health, Faculty of Health Sciences<br>Griffin, Xavier; Queen Mary University of London, Bone and Joint Health, Blizard Institute, ; Royal London Hospital, Barts Health NHS Trust<br>Sohanpal, Ratna; Queen Mary University of London, Centre for Primary Care, Wolfson Institute of Population Health<br>Verkooijen, Helena; University Medical Centre Utrecht; University of Utrecht<br>Maguire, Jonathon; University of Toronto Institute of Health Policy Management and Evaluation<br>McCord, Kimberly; Independent Researcher

**Primary Subject Heading**:	Health services research
Secondary Subject Heading:	Health services research, Ethics, Patient-centred medicine, Public health, Research methods
Keywords:	Clinical Trial, Randomized Controlled Trial, STATISTICS & RESEARCH METHODS, Patient-Centered Care, Decision Making, Patient Participation

SCHOLARONE™
Manuscripts

I, the Submitting Author has the right to grant and does grant on behalf of all authors of the Work (as defined in the below author licence), an exclusive licence and/or a non-exclusive licence for contributions from authors who are: i) UK Crown employees; ii) where BMJ has agreed a CC-BY licence shall apply, and/or iii) in accordance with the terms applicable for US Federal Government officers or employees acting as part of their official duties; on a worldwide, perpetual, irrevocable, royalty-free basis to BMJ Publishing Group Ltd ("BMJ") its licensees and where the relevant Journal is co-owned by BMJ to the co-owners of the Journal, to publish the Work in this journal and any other BMJ products and to exploit all rights, as set out in our licence.

The Submitting Author accepts and understands that any supply made under these terms is made by BMJ to the Submitting Author unless you are acting as an employee on behalf of your employer or a postgraduate student of an affiliated institution which is paying any applicable article publishing charge ("APC") for Open Access articles. Where the Submitting Author wishes to make the Work available on an Open Access basis (and intends to pay the relevant APC), the terms of reuse of such Open Access shall be governed by a Creative Commons licence – details of these licences and which Creative Commons licence will apply to this Work are set out in our licence referred to above.

Other than as permitted in any relevant BMJ Author's Self Archiving Policies, I confirm this Work has not been accepted for publication elsewhere, is not being considered for publication elsewhere and does not duplicate material already published. I confirm all authors consent to publication of this Work and authorise the granting of this licence.

For peer review only

Randomised trials conducted using cohorts: A scoping review

Beverley Nickolls1

Clare Relton1

Lars G. Hemkens2,3,4,5

Merrick Zwarenstein6,7

Sandra Eldridge1

Stephen J. McCall8,9

Xavier Griffin10,11

Ratna Sohanpal12

Helena M. Verkooijen13,14

Jonathon Maguire15

Kimberly A. Mc Cord- De laco16

1. Centre for Evaluation and Methods, Wolfson Institute of Population Health, Queen Mary University of London, UK
2. Research Center for Clinical Neuroimmunology and Neuroscience Basel (RC2NB), University Hospital Basel and University of Basel, Basel, Switzerland
3. Department of Clinical Research, University Hospital Basel and University of Basel, Basel, Switzerland
4. Meta-Research Innovation Center at Stanford (METRICS), Stanford University, Stanford, California, USA
5. Meta-Research Innovation Center Berlin (METRIC-B), Berlin Institute of Health, Berlin, Germany
6. Department of Family Medicine, Western University, London, Canada
7. Institute for Clinical Evaluative Sciences, Toronto, Canada
8. National Perinatal Epidemiology Unit Clinical Trials Unit, Nuffield Department of Population Health, University of Oxford, Oxford, UK
9. Center for Research on Population and Health, Faculty of Health Sciences, American University of Beirut, Ras Beirut, Lebanon
10. Bone and Joint Health, Blizard Institute, Queen Mary University of London, UK
11. Royal London Hospital, Barts Health NHS Trust, London, UK
12. Centre for Primary Care, Wolfson Institute of Population Health, Queen Mary University of London, UK
13. University Medical Center Utrecht, Utrecht, the Netherlands
14. University of Utrecht, Utrecht, the Netherlands
15. Institute of Health Policy, Management and Evaluation, University of Toronto, Canada
16. Independent researcher

For peer review only

Address for Correspondence: Beverley Nickolls, PhD student, Centre for Evaluation and Methods, Wolfson Institute of Population Health, Queen Mary University of London, UK
Email: b.nickolls@qmul.ac.uk

Acknowledgements: We thank Margaret Sampson, MLIS, PhD, AHIP (Children's Hospital of Eastern Ontario, Ottawa, Canada) for developing the search strategies on behalf of the CONSORT team. We thank Dr Philippa Fibert, St Mary's University, Twickenham, London for her help in screening publications for inclusion.

Abstract

Introduction: Cohort studies generate and collect longitudinal data for a variety of research purposes. Randomized controlled trials (RCTs) increasingly use cohort studies as data infrastructures to help identify and recruit trial participants and assess outcomes.

Objective: To examine the extent, range, and nature of research using cohorts for RCTs and describe the varied definitions and conceptual boundaries for RCTs using cohorts.

Design: Scoping review

Data sources: Searches were undertaken in January 2021 in MEDLINE (Ovid) and EBM Reviews—Cochrane Methodology Registry (Final issue, third Quarter 2012).

Eligibility criteria: Reports published between January 2007 and December 2021 of (a) cohorts used or planned to be used, to conduct RCTs, or (b) RCTs which use cohorts to recruit participants and/or collect trial outcomes, or (c) methodological studies discussing the use of cohorts for RCTs.

Data extraction and synthesis: Data were extracted on condition being studied, age group, setting, country/continent, intervention(s) and comparators planned or received, unit of randomisation, timing of randomisation, approach to informed consent, study design, and terminology.

Results: A total of 175 full text articles were assessed for eligibility. We identified 61 protocols, 9 descriptions of stand-alone cohorts intended to be used for future RCTs, 39 RCTs using cohorts, and 34 methodological papers.

The use and scope of this approach is growing. The thematics of study are far-ranging, including population health, oncology, mental and behavioural disorders, and musculoskeletal conditions.

Authors reported this approach can lead to more efficient recruitment, more representative samples, and lessen disappointment bias and crossovers.

Conclusion: This review outlines the development of cohorts to conduct RCTs including the range of use and innovative changes and adaptations. Inconsistencies in the use of terminology and concepts are highlighted. Guidance now needs to be developed to support the design and reporting of RCTs conducted using cohorts.

(296/300 words)

For peer review only - http://bmjopen.bmj.com/site/about/guidelines.xhtml

Strengths and Limitations

- The study's methodological strengths include the use of the CONSORT- ROUTINE extension search strategy to update the scope of work taking place using cohorts.
- Another strength is the overview of methodological writing on trials using cohorts including how informed consent procedures are operationalized.
- While we aimed to assess reporting and terminology, the identification of our sample relied on reporting and terminology.
- The review was limited by the amount of detail available on some important design features e.g. how informed consent processes were operationalised in different clinical populations.
- The studies identified are pre-January 2022. As most are protocols for RCTs yet to run or complete, it is difficult to draw inferences on trends in recruitment and retention. The impact of COVID-19 on these trials is also an unknown factor.

INTRODUCTION

In medical research, the term cohort designates a group of persons who have one or more characteristics in common(1). Cohort studies use longitudinal data obtained from a cohort population to investigate the epidemiology of diseases and establish associations between explanatory factors and health outcomes. Randomised controlled trials (RCTs) create treatment groups through random allocation in order to assess the effects of interventions such as screening, surgery, or drugs. Traditional RCTs are often expensive and time consuming, often rendering designs that are of limited utility including small sample sizes, low generalisability, and lack of long-term outcomes. There is an increasing need for innovative research designs to overcome these shortcomings(2-9).

RCTs conducted within cohorts is an innovative approach which uses new or existing cohort infrastructures and cohort study participants to identify and recruit trial participants and collect outcome data, through the regular measurement of cohort outcomes and procedures(10, 11).

Cohort data infrastructures generate and collect data for the purpose of research and RCTs can be conducted using the cohort infrastructure. RCTs can also use administrative data(12), Electronic Health Records(EHR)(13), and registry data infrastructures(14), i.e. the classic forms of routine data collection. In this scoping review, we cover RCTs using research-based cohorts, but not RCTs using routine, administrative and registry-based data infrastructures which have been described elsewhere(12-15).

Compared with traditional RCTs, there are several advantages to using cohorts to conduct RCTs. If an existing cohort study is used, then the time needed for developing trial recruitment and data

For peer review only - http://bmjopen.bmj.com/site/about/guidelines.xhtml

collection systems can be reduced. RCTs conducted within cohort studies may recruit a more generalisable sample of participants(16). Recruiting trial participants from a cohort provides information on those who decline to participate in the trial. The cohort can also provide ongoing information as to the natural history of the condition and treatment as usual and collect outcomes well beyond the time horizon of many traditional RCTs.

There is variation in how this innovative approach to trial design is named and described. A commonly cited approach is the cohort multiple RCT (cmRCT) design(2); another frequent and more recent term is the Trials within Cohorts (TwiCs) design(11). In this design, participants enrol in an observational cohort study with regular outcome measurement. This provides a framework for the implementation of multiple RCTs. For each RCT embedded in the cohort, a random selection of RCT-eligible patients is contacted and offered the intervention. Outcomes of participants randomly allocated to the intervention group are compared to outcomes of RCT-eligible patients not randomly allocated to the intervention, who receive standard/usual care as defined within the cohort. The information provided to potential trial participants and the consents sought are ‘patient-centred’. The processes in this design aim to replicate informed consent practices as they would be applied in clinical care, i.e. patients are only informed about an intervention if and when they will have access to the intervention(2).

There is variation in how informed consent is operationalised in trials using cohorts. Some RCTs using cohorts use a standard approach to informed consent. In the standard approach, all potential trial participants are informed of all group allocations and also that group allocation will be determined by chance not choice. This information is provided before randomisation. Other trials conducted using cohorts take different approaches to how and when potential and actual participants are given information and their consent sought. A key feature of the TwiCs design is the staging of informed consent. The origins of this approach aimed to be ‘patient-centred’. The ‘staged-consent’ approach(17) further refines this by seeking explicit consent from everyone recruited at the cohort enrolment stage to not be informed in the future if they are randomized to the usual care control group of an RCT.

The timing of randomisation of eligible cohort participants varies. Many trials randomise at one moment in time, using a single-batch sampling approach in their closed or recruiting cohorts. Some use a sequential process whereby individual patients are randomised as soon as they become eligible -which may or may not coincide with diagnosis(18). Given the relative novelty of RCTs using cohorts and the increasing adoption of these designs(1), there is a need to

For peer review only - http://bmjopen.bmj.com/site/about/guidelines.xhtml

understand how this approach is being used in health research. This scoping review supplements the development of the Consolidated Standards of Reporting Trials (CONSORT) extension for the reporting of randomised controlled trials conducted using cohorts and routinely collected data (CONSORT-ROUTINE)(19), which was developed to guide the reporting of the unique characteristics of trials conducted using both research cohorts and three kinds of routinely collected data (EHR, registry, and administrative data)(20).

The primary aim of this review is to examine the extent, range, and nature of research activity for RCTs conducted using cohorts. Given the heterogeneous terminology and overlapping design details that became obvious during the development of reporting guidelines(19), the secondary aim is to clarify working definitions and conceptual boundaries for RCTs using cohorts.

METHODS

The design and reporting of this review follow the PRISMA Extension for Scoping Reviews guidelines(21). The protocol is accessible at Open Science Framework: https://osf.io/ke8pw(22)

A scoping review is defined as 'a form of knowledge synthesis that addresses an exploratory research question aimed at mapping key concepts, types of evidence, and gaps in research related to a defined area or field by systematically searching, selecting, and synthesizing existing knowledge'(23).

Search strategy

The search strategy from the original scoping review that was conducted to support the development of the CONSORT Extension for Trials using Cohorts and Routinely Collected Health Data(20, 24) was updated to cover January 2007 to December 2021. The original search strategy was designed and conducted by an experienced research librarian familiar with knowledge synthesis related to research methods and reporting. This was in collaboration with the CONSORT project team and peer reviewed using the Peer Review of the Electronic Search Strategy (PRESS)(25).

Searches were performed to identify publications describing methodology, trial protocols and results from RCTs that were conducted using cohorts. Searches were undertaken in January 2022 in Ovid MEDLINE Epub Ahead of Print, In-Process & Other Non-Indexed Citations, Ovid MEDLINE Daily and Ovid MEDLINE and EBM Reviews—Cochrane Methodology Registry (Final issue, third Quarter 2012). Searches were conducted covering a fifteen-year period from January 2007- December 2021 which allowed the identification of relatively recent RCTs that

For peer review only - http://bmjopen.bmj.com/site/about/guidelines.xhtml

used cohorts (supplementary file 1). Ten subject experts within the field were contacted to identify new protocols for trials that are being planned and other publications that may have been missed.

The references were imported from the database into Refworks, duplicates removed, and then transferred into DistillerSR (Evidence Partners, Ottawa, Canada). A coding manual based on eligibility criteria was developed (Supplementary file 2). A pilot test of the coding manual was performed prior to the study's inception.

Titles and abstracts were screened independently by two reviewers. A liberal accelerated method was used to identify articles for inclusion in full-text review, where titles and abstracts are screened by one reviewer (BN), and excluded articles are screened by a second reviewer (CR)(26). Full texts were screened independently by two reviewers (BN and PF), and any dissonance was resolved by a third reviewer (CR). At the full-text screening, each reviewer indicated how the cohort was used (used for recruitment, ascertainment of outcomes, both recruitment and outcomes).

Eligibility criteria

Inclusion criteria: any report of cohorts or results of a) cohorts that are used (or planned to be used), to conduct RCTs, b) RCTs within cohorts which use cohorts to recruit participants and/or collect trial outcomes c) methodological studies discussing the use of cohorts for RCTs (Supplementary file 2).

Data extraction

Data were extracted into a Google Documents spreadsheet. For articles reporting primary research the following information was extracted: condition being studied using ISRCTN registry condition categories(27), age group, setting (primary/secondary care/ other), country/ continent, intervention(s) planned or received, unit of randomisation (individual/ cluster) and comparator.

We also extracted data relating to the use of the cohort (participant identification, collection of outcome data), the terminology used to describe the trial design approach, and the approach used to inform and seek consent. Data extraction was completed by a single reviewer (BN) and independently validated by a second reviewer (CR).

Analysis and clarity of reporting

For peer review only - http://bmjopen.bmj.com/site/about/guidelines.xhtml

The reporting of this review followed the guidelines of the Preferred Reporting Items for Systematic Reviews and Meta-Analyses extension for scoping reviews (PRISMA-ScR)(21). The results were reported as a mixture of descriptive numerical synthesis and narrative synthesis. The protocols and results of cohorts and RCTs in cohorts are reported in the tabular analysis and in the narrative synthesis and the methodological articles are described through narrative synthesis of themes for history and development of the terminology and concepts.

Patient and Public Involvement

Although there is no patient and public involvement (PPI) in this scoping review, PPI regarding the design is described in the *Commentary on acceptability of design* section.

RESULTS

The results of the search are presented in Figure 1. Of 2,628 potentially relevant citations, 143 were eligible of which 109 articles were suitable for both quantitative analysis and qualitative synthesis, and 34 methodological articles for qualitative synthesis only.
Of the 61 protocols, the majority (62%, 38/61) described planned cohorts and RCTs together. 18% (11/61) were for protocols for cohorts and 20% (12/61) were for RCTs in cohorts.

Figure 1 PRISMA flowchart (uploaded separately)

1. Research activity for trials conducted using cohorts.

In total 109 publications were reviewed to assess the extent, range, and nature of research activity for trials conducted using cohorts. This included 61 protocols reporting on planned cohorts and/or RCTs, 39 papers reporting RCT results, and 9 papers reporting results of cohort studies established with the facility to host future RCTs. Table 1 reports the characteristics of articles reporting protocols for cohort studies and RCTs in column 1 (green). Articles reporting the results of RCTs embedded in cohorts and the results of cohorts set up hosting RCTs are reported in columns 2 and 3. The references throughout give examples of publications in these three categories. Results are presented as absolute values and percentages.

Table 1: Characteristics of publications reporting protocols for cohorts and results of trials using cohorts.

For peer review only - http://bmjopen.bmj.com/site/about/guidelines.xhtml

For peer review only

	No (%) of PROTOCOLS of cohort studies +/- RCTs) (n/61)		No (%) of RESULTS of RCTs embedded in cohort studies (n/39)		No (%) of RESULTS of cohort studies set up to host future RCTs (n/ 9)	
	n=	%	n=	%	n=	%
**Use of Cohort**						
Participant identification	61	100%	39	100%	9	100%
Outcome data	54	89%	36	92%	9	100%
**Condition being studied**						
Signs & symptoms (population health)	21	34%	10	26%	4	44%
Musculoskeletal diseases	12	20%	8	21%	0	0
Mental and Behavioural disorders	12	20%	3	8%	0	0
Cancer	8	13%	3	8%	3	33%
Infections and infestations	4	7%	3	8%	0	0
Respiratory	4	7%	2	5%	0	0
Urological and genital diseases	3	5%	3	8%	0	0
Nutritional Metabolic, endocrine	2	3%	2	5%	0	0
Cardiovascular	1	2%	3	8%	0	0
Skin and connective tissue diseases	1	2%	0	0	1	11%
Injury, occupational diseases, poisoning	1	2%	0	0	0	0
Ear, nose and throat	0	0	0	0	1	11%
Pregnancy and childbirth	1	2%	5	13%	0	0
Oral health	0	0	2	5%	0	0
Other	0	0	1	3%	0	0
**Age group**						
Adults	46	75%	34	87%	7	78%
Children	9	15%	5	13%	1	11%
Both	6	10%	0	0	1	11%
**Setting**						
Secondary care	40	66%	22	56%	5	56%
Primary care	12	20%	12	31%	4	44%
Other	9	15%	5	13%	0	0
**Country/ Continent**						
Continental Europe	22	36%	15	38%	4	44%
United Kingdom and Ireland	21	34%	13	33%	3	33%
North America	7	11%	3	8%	1	11%
Australasia	5	8%	2	5%	1	11%
Asia	3	5%	3	8%	1	11%
South America	2	3%	0	0%	0	0
Africa	1	2%	3	8%	0	0
**Intervention (s)**						
Behaviour change	17	28%	13	33%	N/A	0
Surgical	13	21%	5	13%	N/A	0
Drug	4	7%	12	31%	N/A	0

For peer review only - http://bmjopen.bmj.com/site/about/guidelines.xhtml

Psychological approaches	9	15%	3	8%	N/A	0
Chemo-radiation	5	8%	2	5%	N/A	0
Complementary therapy	3	5%	4	10%	N/A	0
Physical/Manual therapy	2	3%	5	13%	N/A	0
Screening	2	3%	2	5%	N/A	0
Other	10	16%	5	13%	N/A	0
**Comparator**						
Usual care	50	82%	28	72%	7	78%
Placebo	6	10%	6	15%	0	0
Unclear/Other	5	8%	5	13%	2	22%

Use of cohort

All included studies used cohorts to identify potential participants and almost all 91% (99/109) also used the cohort to obtain some or all outcome data. Of the 39 RCT results papers, almost all (92%, 36/39) described trials which had used cohorts for both recruitment and outcome measurement. Only three RCT results papers reported using cohorts just for recruitment.

Conditions being studied

This section describes the conditions being studied using ISRCTN registry condition categories(27).

The most common category was signs and symptoms (population health and risk prevention), covering almost a third of protocols (22/61), a quarter of articles reporting trial results (10/39), and more than a third of cohort descriptions (4/9). Where there was also a specific disease/condition the study was also placed under this category. For example, a protocol for a population health COVID-19 cohort(28) was placed within both the Signs and Symptoms category and the Respiratory category. A cohort that recruited in pregnancy was categorised as Pregnancy and Childbirth as well as Signs and Symptoms/Population Health(29).

Age groups

Most studies were of adults - 75% (46/61) of protocols, 87% of RCT results (34/39), and 78% of cohort descriptions (7/9). Studies of children included 13% (5/39) of RCT results, 15% of protocols (9/61), and 11% of cohorts (1/9). Only 10% of protocols (6/61) studied both adults and children. These included planned cohorts for both parents and children (29, 30) and a trial that straddled the child/adult threshold (31).

For peer review only - http://bmjopen.bmj.com/site/about/guidelines.xhtml

Settings

The most common setting was secondary care with 66% of protocols (40/61), 56% of RCTs (22/39), and 56% of cohorts (5/9). Primary care settings accounted for 20% of protocols (12/61), 31% of RCTs (12/39), and 44% (4/9) of cohorts. The remaining studies described RCTs 15% (9/61) and protocols 13% (5/39) in community(32) and educational(33, 34) settings.

Country/Continent

The majority of publications came from Continental Europe, with 36% (22/61) protocols, 38% (15/39) RCTs, and 44% (4/9) stand-alone cohorts. This was closely followed by the United Kingdom and Ireland, with contributions from North America, Australasia, Asia, South America, and Africa.

Interventions

The most common interventions studied were behaviour change with 28% (17/61) of protocols and 33% (13/39) of RCTs. Some population health based cohorts planned to identify interventions after the cohort study had started data collection and thus were unable to identify which interventions would be tested e.g.(29, 30, 35, 36)). Some interventions fell into two or more categories, either because it could be classed as both, for example, behavioural change and psychological approaches, e.g.(37), or because the trial combined different categories of interventions, e.g. complementary therapy and nutrition e.g.(38). Some interventions were categorised as 'other', e.g. helmet therapy(39), cough treatment algorithms(40), and nurse-led medicine monitoring(41).

Comparators

Most studies included treatment as usual (TAU) comparators - 82% (50/61) of protocols, 72% (28/39) of RCTs, and 78% (7/9) of cohorts as defined within the cohort. TAU is assumed to refer to unrestricted usual care with no intervention, restriction or standardisation placed on the clinician or recipient, but this was not clarified in some studies. Placebo was used in 10% (6/61) of protocols(42, 43) and 15% (6/39) of RCTs(44-46). The 'other' category included an active comparator(47).

In several cohorts it was not stated what the trial comparators would be, e.g.(48) but as expected, all RCTs using the staged approach had a usual care comparator. In the results of stand-alone

For peer review only - http://bmjopen.bmj.com/site/about/guidelines.xhtml

cohorts intended to be used for RCTs, we categorised them as having a usual care comparator if the publication made reference to using a staged or person-centred approach to informed consent.

2. **Terminology and Conceptual Boundaries**

In addition to publications reporting research activity (Table 1), there were 34 methodology papers (Table 2). These were analysed to explore the development of the terminology and concepts and contribute to the qualitative synthesis. This table shows the type of trial design, approach to informed consent, terminology used to describe the type of trial design, and timing of randomisation to an RCT. The protocols are reported together in column 1 and the results of the RCTs and cohorts for hosting RCTs are given in columns 2 and 3.

Table 2: Methodological Papers

Focus of Paper	Numbers (n/34)		First author and Reference
	n	%	
Cohort designs for clinical areas	6	18	Ahmed(49), Couwenberg(18), Gal(50), Heaven(51), Young-Afat(52) Zakrzewska(53)
CmRCT/TwiCs design	17	50	Bibby(54), Candlish(55), Clegg(56), Flory(57), Kim(58), Lambin(59), Pate(60), Relton(2, 11), Reeves(61) Richards(54), Van der Velden(62), Verkooijen(63), Verweij(64), Vickers(65) Viksveen(16), Young-Afat(17)
Learning Health Systems (using TwiCs)	1	3	Wouters(66)
Studies within a Trial (SWATs) using cohorts	10	30	Arundel(67), Cockayne(68), Cotterill(69), Boyd(70), Goodwin(71), Knapp(72), Ni(73), Loban(74), Maruani ((75) Wakabayashi(76),

Type of trial design

The majority of protocols and trials reports described individually randomised RCTs. Cluster trial designs were described in 8% (5/61) of protocols and trial reports (3/39) and stepped wedge designs in 3% (2/61) of protocols and 5% (2/39) of trial reports. Table 3 reports the characteristics of articles reporting protocols for cohort studies and RCTs in column 1 (green). Articles reporting the results of RCTs embedded in cohorts and the results of cohorts set up hosting RCTs are reported in columns 2 and 3. The references throughout give examples of publications in these three categories. Results are presented as absolute values and percentages.

For peer review only - http://bmjopen.bmj.com/site/about/guidelines.xhtml

Table 3:Terminology and Conceptual Boundaries in articles reporting protocols for cohorts, and results of trials using cohorts.

	No (%) of PROTOCOLS of cohort studies +/- trials (n/61)		No (%) of RESULTS of RCTs embedded in cohort studies (n/39)		No (%) of RESULTS of cohort studies set up to host future RCTs (n/9)	
	n=	%	n=	%	n=	%
**Trial design**						
Individually randomised	54	89%	34	87%	7	78%
Cluster randomised	5	8%	3	8%	0	0
Stepped wedge	2	3%	2	5%	0	0
Not known	0	0%	0	0	2	22%
**Informed consent approach**						
'Patient-centred'/'Staged-consent'	32	52%	12	31%	7	78%
'All Upfront' standard approach	19	31%	21	54%	0	0
Not described	2	3%	2	5%	0	0
Other	8	13%	4	10%	0	0
**Design terminology used**						
Cohort multiple RCT (cmRCT)	16	26%	6	15%	5	56%
Trials within Cohorts (TwiCs)	12	20%	5	13%	1	11%
Cohort-embedded RCT	9	15%	12	31%	0	0
Cohort-nested RCT	11	18%	4	10%	0	0
Other	13	21%	12	31%	3	33%
**Timing of randomisation to RCT**						
Sequential randomisation	34	56%	15	38%	0	0
Batch randomisation	20	33%	23	59%	0	0
Not stated/known	7	11%	1	3%	9	100%

Approach to informed consent

Some studies stated whether they were using either a 'patient-centred' or 'staged' approach to informed consent, either by using these terms or by citing Young-Afat(17) 'Staged consent' publication or Relton(2) when describing their approach.

Over half (52%) of protocols (32/61) and 31% (12/39) of trials reported using a staged approach. Studies not using this approach included cluster trials(34, 77-80).

Terminology

The term cohort multiple RCT (cmRCT) was used in 26% (16/61) of protocols and 15% (6/39) of RCTs. Trials within Cohorts (TwiCs) was used in 20% (12/61) of protocols and 13% (5/39) of trials. Cohort-embedded RCT was used in 15% (9/61) of protocols and 31% (12/39) of trials. Other terms used include cohort-nested RCT, cohort RCT, and cohort study.

For peer review only - http://bmjopen.bmj.com/site/about/guidelines.xhtml

In relation to informed consent, there were 68 publications that used or described ‘patient-centred’ or staged approaches. Publications were identified if they either a) used the term ‘patient-centred’ or ‘staged’ or cited Young-Afat(17) or Relton(2) when describing their approach. One study cites using a staged approach but does not use any terminology to describe the design of its future RCTs(81). The terminology used in the 67 remaining publications is reported in Table 4.

Table 4 reports the terminology of studies using staged informed consent in methodological publications and publications reporting research activity. This table shows the terminology for methodological literature, protocols, and RCTs using cohorts. The reference to each publication is given and the colour index is given below the table. The terms cmRCT, cohort-embedded, and cohort-nested are given from 2010 when the patient-centred approach to IC was first described, and the term TwiCs from 2016 when it was first coined in the literature. Cohort-embedded and cohort-nested are given together with the codes ‘em’ for embedded and ‘n’ for nested.

For peer review only

For peer review only - http://bmjopen.bmj.com/site/about/guidelines.xhtml

Table 4:Terminology used to describe trial designs for RCTs with a patient-centred/staged approach to IC.

Methodological literature (n=24) Protocols (n=31). RCTs using cohorts (n=12)						
Trial design/ Year	2010 - 2011	2012 - 2013	2014 - 2015	2016 - 2017	2018 - 2019	2020 - 2021
cmRCT	(2)	(63)	(54)<br>(56, 59)	(17, 51, 53, 57, 60)<br>(16, 55, 82)	(61)	
	(83),(35)	(84)<br>(85)	(86, 87)<br>(31, 88)	(89, 90)<br>(91)	(92, 93)	
		(94)	(95)	(96, 97)	(98-100)	
TwiCs				(11)	(58, 61, 101)	(18, 66)<br>(52, 64, 102, 103)
				(29, 104)	(33)<br>(105-107)	(30, 108-111)<br>(112-114)
					(37, 115)<br>(38)	(116, 117)
Cohort-embedded/ nested RCT			(49)			
		(118)	(119)	(40)		(120)

Table 4 Index:
Methodological literature (n=24)
Protocol (n=31)
RCTs using cohorts (n=12)

From 2018 onwards there is a noticeable shift in the terminology used in protocols, with the majority of protocols describing Trials within Cohorts (TwiCs), a name that was coined at the 2016 Ethics of TwiCs Symposium(11). This shift in terminology was also seen in the methodological literature, where 100% (9/9) papers identified from 2018-2021 refer to the design as Trials within Cohorts, with one paper using both terms TwiCs and cmRCT(61).

Timing of randomisation

Fifty-six per cent (34/61) of the protocols and 38% (15/39) of the RCTs stated that they would or had used sequential randomisation, compared to batch randomisation which was used by 33% (20/61) of protocols and 59% (23/39). The timing of randomisation was not clear in 11% of protocols and 3% of RCTs.

DISCUSSION

Three main areas emerged from the narrative synthesis of themes arising in the studies and methodological literature: commentary on efficiency of recruitment, commentary on acceptability, and new conceptual developments. These are discussed below:

Commentary on efficiency of recruitment

Unusually, no trials reported being unable to recruit to time and target(121).

Most articles commented on the efficiency of the design, enhanced recruitment, and the potential to embed in multiple RCTs. Young-Afat et al(122) reported that 88% of patients invited to join the UMBRELLA cohort consented to participate, most of whom (88%) gave broad consent for randomisation to future interventions. Coebergh van den Braak et al(123) proposed that the efficiency of this approach arises from physicians saving time by only explaining an intervention that they can *actually* offer to a patient. Viksveen et al(97) reported that the trial '*over-recruited*', and how the design enabled the acceptability of an intervention to be measured by intervention take-up in the intervention group. Viksveen et al(16) also argued that this approach has the potential to recruit more representative populations than stand-alone recruitment to an RCT and that the '*main benefits of using the cmRCT design to test an intervention for self-reported depression were full, fast and efficient recruitment; lower attrition rates than other depression trials; and a trial population broadly similar to the general population of patients self-reporting chronic moderate to severe depression*'(16).

For peer review only - http://bmjopen.bmj.com/site/about/guidelines.xhtml

Most trials (33/39) and all protocols stated that they had or would use an Intention to Treat (ITT) analysis as part of their statistical approach. Some also recommended using a complier average causal effect (CACE) analysis instead of per protocol analysis due to risk of bias(16, 97). In CACE analyses, outcomes in patients who take up the offer of treatment are compared to outcomes in the no offer group of patients who would have taken up the offer had they received it. Some authors used instrumental variables (IV) analysis, as this type of CACE-analysis takes into account patients' baseline characteristics(16, 37, 62, 68, 94), and commented that this analysis was appropriate where there is lower take-up in the intervention arm, especially where the intervention is the offer of treatment.

One publication (Gal et al(50)) compared recruitment to a standalone RCT to recruitment from within a stage-consenting cohort. Recruitment from the stage-consenting cohort was significantly cheaper and faster and prevented contamination between groups '*but noncompliance due to refusal of the intervention was higher compared with conventional pragmatic exercise oncology RCTs, which may dilute the estimated intervention effect'*.

Commentary on acceptability of design

Several studies described Patient and Public Involvement (PPI) work around the trial design in the field of prostate cancer research (49) and kidney disease (45). An online survey conducted in collaboration with the charity Kidney Cancer UK to ascertain acceptability of the study's design and the willingness of patients to be recruited into the cohort found *'strong support and need from the kidney cancer patient community for the broad concepts of the study'*.

The first published evaluations of broad informed consent for randomisation within a TwiCs study were conducted in the Netherlands (52, 64). Cancer patients participating in ongoing TwiCs(52) and a separate study were surveyed between two to six years after cohort entry(64). The approach was found to be generally well accepted by patients with only 2% of patients in the trial usual care control group stating that they would feel negative about not being selected for interventions while their data are being used as a comparator. Both evaluations support the use of the TwiCs design with the staged-informed consent procedure.

New conceptual developments

Most cohorts using the TwiCs design approach were of clinical populations. Some publications described population cohorts e.g. England's 'Born in Bradford Better Start' (BIBBS) cohort (29)

For peer review only - http://bmjopen.bmj.com/site/about/guidelines.xhtml

and the Australian 'Generation Victoria' population cohort (30). Some publications described '*multi-nationa*l' cohorts - the Scleroderma Patient-centred Intervention Network (SPIN) cohort which involves 42 centres globally(85), the Emotional Competence in Young Adults (Ecoweb) PROMOTE platform to promote mental health in young adults (110) which uses a web-based multi-national recruitment strategy, and the European Prevention of Alzheimer's Dementia Longitudinal Cohort Study (EPAD LCS)(81). The EPAD Project is a large collaboration supporting a platform for the testing of multiple interventions. Solomon et al describe how *'As the EPAD project is multistaged, staged consent will be used as a decision-making model. Staged consent feeds relevant information—bit by bit, extended over time—to participants and study partners, and asks informed consent at every step when they need to make important decisions'(81).*

This review reports the characteristics of trials conducted using cohorts and observed how terminology and conceptual boundaries evolved over time in the methodological literature and the literature reporting research activity.

A diverse range of cohorts and trials using cohorts in a wide range of populations were identified. The TwiCs design approach is being utilised for a wide range of clinical conditions and interventions. A major clinical group is oncology (89, 108, 116, 124) in the Netherlands. Use of the TwiCs design is spreading with innovative work taking place in a range of locations and new collaborations are leading to the development of multi-national recruitment.

The term 'staged' approach to informed consent coined in 2016(17) has replaced the earlier term 'patient-centred' in the majority of studies.

All the RCTs using the TwiCs design had usual care for at least one of the comparators - a prerequisite to using a staged approach, i.e. not to contact cohort participants further in the usual care group if they are not selected for trial interventions.

Either batch or sequential randomisation can be utilised as part of a TwiCs design depending on clinical condition and demands of study. However, the majority of protocols use sequential randomisation, where individuals are randomised as they become eligible for the experimental intervention (18).

The TwiCs approach has been cited as an example of an '*Optimization learning health care system*'(66) for encompassing strategies that contribute to a health care system in which *"the*

For peer review only - http://bmjopen.bmj.com/site/about/guidelines.xhtml

process of generating and applying the best evidence will be natural and seamless components of the process of care itself." Use of a staged informed consent process, routine data, and individual randomisation at the point of diagnosis could all be considered examples of these strategies.

To-date the acceptability work around the TwiCs design has been mainly conducted in the field of oncology. Further work to assess the impact for different clinical groups is recommended. There is also a lack of direct comparison with standard RCTs.

This review confirms findings in the earlier scoping review conducted by the CONSORT-ROUTINE team(19) that there is heterogeneity in the terminology and conceptual boundaries for the use of cohorts for RCTs. The term TwiCs is now used for the majority of studies but there is still some variation in terminology and use of the design. There are no current guidelines to support consistency of use.

Methodological limitations

While we aimed to assess reporting and terminology, the identification of our sample relied on reporting and terminology. The review was limited by the amount of detail available on some important design features e.g. how informed consent processes were operationalised in different clinical populations.

As all the studies identified are pre-January 2022 and most are protocols rather than completed studies, it is difficult to draw inferences on trends in recruitment and retention. Moreover, the impact of the COVID-19 pandemic on these trials is unknown.

CONCLUSION

This review identified increasing sophistication and maturity in RCTs conducted within cohorts in a wide range of conditions and research studies including 'mega-cohorts'(30). Our findings support the increased use of the TwiCs design to address some of the barriers to recruitment and retention for standard RCTs with usual care comparators. Further research into the acceptability, use, and efficiency of staged approaches to informed consent, compared to standard approaches, is required. We recommend the use of the term TwiCs to unify approaches and call for an international symposium to define terminology and develop guidelines for best practice for studies using the TwiCs approach.

4,334 words

For peer review only - http://bmjopen.bmj.com/site/about/guidelines.xhtml

Contributor Statement

The conception and design of this work, acquisition of data, initial analysis and preparation of each draft have been the responsibility of BN as part of her PhD studies, supported by CR, XG and RS. CR, LH, MZ, SE, SM, XG, RS, HV, JM and KM have contributed substantially to the analysis and interpretation of the work, reviewing each draft critically for important intellectual content. BN, CR, LH, MZ, SE, SM, XG, RS, HV, JM and KM are jointly responsible for final approval of the version to be published and agreement to be accountable for all aspects of the work in ensuring that questions related to the accuracy or integrity of any part of the work are appropriately investigated and resolved.

Data Availability Statement

The electronic search strategies and inclusion and exclusion criteria are uploaded as supplementary information. References to all studies using a staged approach to informed consent are given in Table 4. A full data set is available on reasonable request.

Funding Statement

We have not received a specific grant from any funding agency in the public, commercial or not-for-profit sectors to conduct this research. Publication costs for this article have been met by Queen Mary University of London under the BMJ Open Access publication agreement.

Competing Interests Statement

Publication costs for this article have been met by Queen Mary University of London under the BMJ Open Access publication agreement.

Ethics Approval Statement

Ethics approval is not required for this scoping review.

For peer review only

References

1. Barría RM. Introductory Chapter: The Contribution of Cohort Studies to Health Sciences. Cohort Studies in Health Sciences: InTech; 2018.
2. Relton C, Torgerson D, Cathain AO, Nicholl J. Rethinking pragmatic randomised controlled trials: introducing the "cohort multiple randomised controlled trial" design. BMJ. 2010;340:c1066.
3. Bower P, Brueton V, Gamble C, Treweek S, Smith CT, Young B, et al. Interventions to improve recruitment and retention in clinical trials: a survey and workshop to assess current practice and future priorities. Trials. 2014;15:399.
4. Watson JM, Torgerson DJ. Increasing recruitment to randomised trials: a review of randomised controlled trials. BMC Medical Research Methodology. 2006;6(1).
5. Campbell M, Snowdon C, Francis D, Elbourne D, Mcdonald A, Knight R, et al. Recruitment to randomised trials: strategies for trial enrolment and participation study. The STEPS study. Health Technology Assessment. 2007;11(48).
6. Treweek S, Lockhart P, Pitkethly M, Cook JA, Kjeldstrøm M, Johansen M, et al. Methods to improve recruitment to randomised controlled trials: Cochrane systematic review and meta-analysis. BMJ Open. 2013;3(2):e002360.
7. Sully BGO, Julious SA, Nicholl J. A reinvestigation of recruitment to randomised, controlled, multicenter trials: a review of trials funded by two UK funding agencies. Trials. 2013;14(1):166.
8. Mcdonald AM, Treweek S, Shakur H, Free C, Knight R, Speed C, et al. Using a business model approach and marketing techniques for recruitment to clinical trials. Trials. 2011;12(1):74.
9. Donovan JL, Paramasivan S, De Salis I, Toerien M. Clear obstacles and hidden challenges: understanding recruiter perspectives in six pragmatic randomised controlled trials. Trials. 2014;15(1):5.
10. Relton C, Thomas K, Nicholl J, Uher R. Review of an innovative approach to practical trials: the 'cohort multiple RCT' design. Trials. 2015;16(S2).
11. Relton C, Burbach M, Collett C, Flory J, Gerlich S, Holm S, et al. The ethics of 'Trials within Cohorts' (TwiCs): 2nd international symposium. Trials. 2017;18(S2).
12. Imran M, Mc Cord K, Mccall SJ, Kwakkenbos L, Sampson M, Fröbert O, et al. Reporting transparency and completeness in trials: Paper 3 – trials conducted using administrative databases do not adequately report elements related to use of databases. Journal of Clinical Epidemiology. 2022;141:187-97.
13. Mccall SJ, Imran M, Hemkens LG, Mc Cord K, Kwakkenbos L, Sampson M, et al. Reporting transparency and completeness in trials: Paper 4 - reporting of randomised controlled trials conducted using routinely collected electronic records – room for improvement. Journal of Clinical Epidemiology. 2022;141:198-209.
14. Mc Cord KA, Imran M, Rice DB, Mccall SJ, Kwakkenbos L, Sampson M, et al. Reporting transparency and completeness in Trials: Paper 2 - reporting of randomised trials using registries was often inadequate and hindered the interpretation of results. Journal of Clinical Epidemiology. 2021.
15. Mc Cord KA, Al-Shahi Salman R, Treweek S, Gardner H, Strech D, Whiteley W, et al. Routinely collected data for randomized trials: promises, barriers, and implications. Trials. 2018;19(1).
16. Viksveen P, Relton C, Nicholl J. Benefits and challenges of using the cohort multiple randomised controlled trial design for testing an intervention for depression. Trials [Electronic Resource]. 2017;18(1):308.
17. Young-Afat DA, Verkooijen HM, Gils CHV, er V, Burbach J, Elias SG, et al. Staged-informed consent in the cohort multiple randomized controlled trial design. Epidemiology. 2016;2016 Jan 06.
18. Couwenberg AM, Burbach JPM, May AM, Berbee M, Intven MPW, Verkooijen HM. The trials within cohorts design facilitated efficient patient enrollment and generalizability in oncology setting. Journal of Clinical Epidemiology. 2020;120:33-9.

For peer review only - http://bmjopen.bmj.com/site/about/guidelines.xhtml

19. Kwakkenbos L, Imran M, Mccall SJ, Mccord KA, Fröbert O, Hemkens LG, et al. CONSORT extension for the reporting of randomised controlled trials conducted using cohorts and routinely collected data (CONSORT-ROUTINE): checklist with explanation and elaboration. BMJ. 2021:n857.
20. Kwakkenbos L, Juszczak E, Hemkens LG, Sampson M, Frobert O, Relton C, et al. Protocol for the development of a CONSORT extension for RCTs using cohorts and routinely collected health data. Res Integr Peer Rev. 2018;3:9.
21. Tricco AC, Lillie E, Zarin W, O'Brien KK, Colquhoun H, Levac D, et al. PRISMA Extension for Scoping Reviews (PRISMA-ScR): Checklist and Explanation. Annals of Internal Medicine. 2018;169(7):467.
22. Nickolls B, Relton C, Eldridge S, Zwarenstein M, McCall S, Hemkens L, et al. Protocol for a scoping review of randomised controlled trials conducted using cohorts. Open Science Framework. 2021.
23. Colquhoun HL, Levac D, O'Brien KK, Straus S, Tricco AC, Perrier L, et al. Scoping reviews: time for clarity in definition, methods, and reporting. Journal of Clinical Epidemiology. 2014;67(12):1291-4.
24. Kwakkenbos L, Imran M, McCord KA, Sampson M, Frobert O, Gale C, et al. Protocol for a scoping review to support development of a CONSORT extension for randomised controlled trials using cohorts and routinely collected health data. BMJ Open. 2018;8(8):e025266.
25. Mcgowan J, Sampson M, Salzwedel DM, Cogo E, Foerster V, Lefebvre C. PRESS Peer Review of Electronic Search Strategies: 2015 Guideline Statement. Journal of Clinical Epidemiology. 2016;75:40-6.
26. Khangura S, Konnyu K, Cushman R, Grimshaw J, Moher D. Evidence summaries: the evolution of a rapid review approach. Systematic Reviews. 2012;1(1):10.
27. ISRCTN. Registry Definitions: Biomed Central Ltd 2020 [cited 2021 January 12th]. Available from: http://www.isrctn.com/page/definitions.
28. Martineau PA. COVIDENCE UK - National longitudinal Study of COVID-19. SMD COVID Research Seminar Series. UK: Queen Mary University London; 2020.
29. Dickerson J, Bird PK, McEachan RR, Pickett KE, Waiblinger D, Uphoff E, et al. Born in Bradford's Better Start: an experimental birth cohort study to evaluate the impact of early life interventions. BMC Public Health. 2016;15:711.
30. Wake M, Hu YJ, Warren H, Danchin M, Fahey M, Orsini F, et al. Integrating trials into a whole-population cohort of children and parents: statement of intent (trials) for the Generation Victoria (GenV) cohort. BMC Medical Research Methodology. 2020;20(1).
31. Snider C, Jiang D, Logsetty S, Strome T, Klassen T. Wraparound care for youth injured by violence: study protocol for a pilot randomised control trial. BMJ Open. 2015;5(5):e008088-e.
32. Finucane FM, Sharp SJ, Purslow LR, Horton K, Horton J, Savage DB, et al. The effects of aerobic exercise on metabolic risk, insulin sensitivity and intrahepatic lipid in healthy older people from the Hertfordshire Cohort Study: a randomised controlled trial. Diabetologia. 2010;53(4):624-31.
33. Fibert P, Relton C, Peasgood T, Daley D. Protocol for the STAR (Sheffield Treatments for ADHD) project: an internal pilot study assessing the feasibility of the Trials within Cohorts (TwiCs) design to test the effectiveness of interventions for children with ADHD. Pilot Feasibility Stud. 2018;4:61.
34. Hafkamp-De Groen E, Mohangoo AD, De Jongste JC, Van Der Wouden JC, Moll HA, Jaddoe VW, et al. Early detection and counselling intervention of asthma symptoms in preschool children: study design of a cluster randomised controlled trial. BMC Public Health. 2010;10(1):555.
35. Relton C, Bissell P, Smith C, Blackburn J, Cooper CL, Nicholl J, et al. South Yorkshire Cohort: a 'cohort trials facility' study of health and weight - protocol for the recruitment phase. BMC Public Health. 2011;11:640.
36. Finer S, Martin HC, Khan A, Hunt KA, Maclaughlin B, Ahmed Z, et al. Cohort Profile: East London Genes & Health (ELGH), a community-based population genomics and health study in British Bangladeshi and British Pakistani people. International Journal of Epidemiology. 2020;49(1):20-1i.
37. Panagioti M, Reeves D, Meacock R, Parkinson B, Lovell K, Hann M, et al. Is telephone health coaching a useful population health strategy for supporting older people with multimorbidity? An evaluation of reach, effectiveness and cost-effectiveness using a ‘trial within a cohort’. BMC Medicine. 2018;16(1).

For peer review only - http://bmjopen.bmj.com/site/about/guidelines.xhtml

38. Fibert P, Peasgood T, Relton C. Rethinking ADHD intervention trials: feasibility testing of two treatments and a methodology. Eur J Pediatr. 2019.
39. van Wijk RM, van Vlimmeren LA, Groothuis-Oudshoorn CG, Van der Ploeg CP, Ijzerman MJ, Boere-Boonekamp MM. Helmet therapy in infants with positional skull deformation: randomised controlled trial. BMJ. 2014;348:g2741.
40. Grady KFO, Grimwood K, Toombs M, Sloots TP, Otim M, Whiley D, et al. Effectiveness of a cough management algorithm at the transitional phase from acute to chronic cough in Australian children aged <15 years: protocol for a randomised controlled trial. BMJ Open. 2017;7(3):e013796.
41. Jordan S, Gabe-Walters ME, Watkins A, Humphreys I, Newson L, Snelgrove S, et al. Nurse-Led Medicines' Monitoring for Patients with Dementia in Care Homes: A Pragmatic Cohort Stepped Wedge Cluster Randomised Trial. PLoS One. 2015;10(10):e0140203.
42. Beekman E, Mesters I, Hendriks EJ, Muris JW, Wesseling G, Evers SM, et al. Exacerbations in patients with chronic obstructive pulmonary disease receiving physical therapy: a cohort-nested randomised controlled trial. BMC Pulmonary Medicine. 2014;14:71.
43. Sihvonen R, Paavola M, Malmivaara A, Jarvinen TL. Finnish Degenerative Meniscal Lesion Study (FIDELITY): a protocol for a randomised, placebo surgery controlled trial on the efficacy of arthroscopic partial meniscectomy for patients with degenerative meniscus injury with a novel 'RCT within-a-cohort' study design. BMJ Open. 2013;3(3).
44. Tofte N, Lindhardt M, Adamova K, Bakker SJL, Beige J, Beulens JWJ, et al. Early detection of diabetic kidney disease by urinary proteomics and subsequent intervention with spironolactone to delay progression (PRIORITY): a prospective observational study and embedded randomised placebo-controlled trial. The Lancet Diabetes & Endocrinology. 2020;8(4):301-12.
45. Freedman DM, Kuncl RW, Weinstein SJ, Malila N, Virtamo J, Albanes D. Vitamin E serum levels and controlled supplementation and risk of amyotrophic lateral sclerosis. Amyotrophic Lateral sclerosis & Frontotemporal Degeneration. 2013;14(4):246.
46. Kazemier BM, Koningstein FN, Schneeberger C, Ott A, Bossuyt PM, de Miranda E, et al. Maternal and neonatal consequences of treated and untreated asymptomatic bacteriuria in pregnancy: a prospective cohort study with an embedded randomised controlled trial. Lancet Infect Dis. 2015;15(11):1324-33.
47. Sheeran L, Deursen Rv, Caterson B, Sparkes V. Classification-guided versus generalized postural intervention in subgroups of nonspecific chronic low back pain: a pragmatic randomized controlled study. Spine. 2013;38(19):1613.
48. Renteln-Kruse Wv, Dapp U, Anders J, Profener F, Schmidt S, Deneke C, et al. The LUCAS* consortium: objectives of interdisciplinary research on selected aspects of ageing and health care for older people in an urban community. Zeitschrift fur Gerontologie und Geriatrie. 2011;44(4):250.
49. Ahmed HU, Berge V, Bottomley D, Cross W, Heer R, Kaplan R, et al. Can we deliver randomized trials of focal therapy in prostate cancer?. [Review][Erratum appears in Nat Rev Clin Oncol. 2017 Sep 12;:; PMID: 28895571]. Nature Reviews Clinical Oncology. 2014;11(8):482.
50. Gal R, Monninkhof EM, Groenwold RHH, Van Gils CH, Van Den Bongard DHJG, Peeters PHM, et al. The effects of exercise on the quality of life of patients with breast cancer (the UMBRELLA Fit study): study protocol for a randomized controlled trial. Trials. 2017;18(1).
51. Heaven A, Brown L, Foster M, Clegg A. Keeping it credible in cohort multiple Randomised Controlled Trials: the Community Ageing Research 75+ (CARE 75+) study model of patient and public involvement and engagement. Research Involvement & Engagement. 2016;2:30.
52. Young-Afat DA, Gal R, Gerlich S, Burbach JPM, Van Der Velden JM, Van Den Bongard DHJG, et al. Oncology patients were found to understand and accept the Trials within Cohorts design. Journal of Clinical Epidemiology. 2021;130:135-42.
53. Zakrzewska JM, Relton C. Future Directions for Surgical Trial Designs in Trigeminal Neuralgia. [Review]. Neurosurgery Clinics of North America. 2016;27(3):353.

For peer review only - http://bmjopen.bmj.com/site/about/guidelines.xhtml

54. Richards DA, Ross S, Robens S, Borglin G. The DiReCT study - improving recruitment into clinical trials: a mixed methods study investigating the ethical acceptability, feasibility and recruitment yield of the cohort multiple randomised controlled trials design. Trials [Electronic Resource]. 2014;15:398.
55. Candlish J, Pate A, Sperrin M, Staa Tv, Work Package G. Evaluation of biases present in the cohort multiple randomised controlled trial design: a simulation study.[Erratum appears in BMC Med Res Methodol. 2017 Mar 22;17 (1):47; PMID: 28330450]. BMC Medical Research Methodology. 2017;17(1):17.
56. Clegg A, Relton C, Young J, Witham M. Improving recruitment of older people to clinical trials: use of the cohort multiple randomised controlled trial design. Age Ageing. 2015;44(4):547-50.
57. Flory JH, Mushlin AI, Goodman ZI. Proposals to Conduct Randomized Controlled Trials Without Informed Consent: a Narrative Review. [Review]. Journal of General Internal Medicine. 2016;31(12):1511.
58. Kim SY, Flory J, Relton C. Ethics and practice of Trials within Cohorts: An emerging pragmatic trial design. Clin Trials. 2018;15(1):9-16.
59. Lambin P, Zindler J, Vanneste B, e v, Jacobs M, Eekers D, et al. Modern clinical research: How rapid learning health care and cohort multiple randomised clinical trials complement traditional evidence based medicine. Acta Oncologica. 2015;54(9):1289.
60. Pate A, Candlish J, Sperrin M, Staa TPV, Work Package G. Cohort Multiple Randomised Controlled Trials (cmRCT) design: efficient but biased? A simulation study to evaluate the feasibility of the Cluster cmRCT design. BMC Medical Research Methodology. 2016;16(1):109.
61. Reeves D, Howells K, Sidaway M, Blakemore A, Hann M, Panagioti M, et al. The cohort multiple randomized controlled trial design was found to be highly susceptible to low statistical power and internal validity biases. Journal of Clinical Epidemiology. 2018;95:111.
62. van der Velden JM, Verkooijen HM, Young-Afat DA, Burbach JP, van Vulpen M, Relton C, et al. The cohort multiple randomized controlled trial design: a valid and efficient alternative to pragmatic trials? Int J Epidemiol. 2017;46(1):96-102.
63. Verkooijen HM, Roes K, van Gils CH. [Cohort multiple randomized controlled trial: a solution for the evaluation of multiple interventions]. Ned Tijdschr Geneeskd. 2013;157(17):A5762.
64. Verweij ME, Gal R, Burbach JPM, Young-Afat DA, Van Der Velden JM, Van Der Graaf R, et al. Most patients reported positively or neutrally of having served as controls in the trials within cohorts design. Journal of Clinical Epidemiology. 2022;148:39-47.
65. Vickers AJ, Young-Afat DA, Ehdaie B, Kim SY. Just-in-time consent: The ethical case for an alternative to traditional informed consent in randomized trials comparing an experimental intervention with usual care. Clin Trials. 2018;15(1):3-8.
66. Wouters. Learning health care systems: Highly needed but challenging. Wiley Online Library. 2020.
67. Arundel C, Jefferson L, Bailey M, Cockayne S, Hicks K, Loughrey L, et al. A randomized, embedded trial of pre-notification of trial participation did not increase recruitment rates to a falls prevention trial. Journal of Evaluation in Clinical Practice. 2017;23(1):73.
68. Cockayne S, Adamson J, Clarke A, Corbacho B, Fairhurst C, Green L, et al. Cohort Randomised Controlled Trial of a Multifaceted Podiatry Intervention for the Prevention of Falls in Older People (The REFORM Trial). PLoS ONE [Electronic Resource]. 2017;12(1):e0168712.
69. Cotterill S, Howells K, Rhodes S, Bower P. The effect of using social pressure in cover letters to improve retention in a longitudinal health study: an embedded randomised controlled retention trial. Trials. 2017;18(1).
70. Boyd A, Tilling K, Cornish R, Davies A, Humphries K, Macleod J. Professionally designed information materials and telephone reminders improved consent response rates: evidence from an RCT nested within a cohort study. Journal of Clinical Epidemiology. 2015;68(8):877.
71. Goodwin M, Walsh T, Whittaker W, Emsley R, Sutton M, Tickle M, et al. Increasing questionnaire response: evidence from a nested RCT within a longitudinal birth cohort study. BMC Medical Research Methodology. 2020;20(1).

For peer review only - http://bmjopen.bmj.com/site/about/guidelines.xhtml

72. Knapp P, Raynor DK, Silcock J, Parkinson B. Can user testing of a clinical trial patient information sheet make it fit-for-purpose? - a randomized controlled trial. BMC Medicine. 2011;9(1):89.
73. Ni MY, Li TK, Hui RWH, McDowell I, Leung GM. Requesting a unique personal identifier or providing a souvenir incentive did not affect overall consent to health record linkage: evidence from an RCT nested within a cohort. Journal of Clinical Epidemiology. 2017;84:142.
74. Loban A, Mandefield L, Hind D, Bradburn M. A randomized trial found online questionnaires supplemented by postal reminders generated a cost-effective and generalizable sample but don't forget the reminders. Journal of Clinical Epidemiology. 2017;92:116-25.
75. Maruani A, Boutron I, Baron G, Ravaud P. Impact of sending email reminders of the legal requirement for posting results on ClinicalTrials.gov: cohort embedded pragmatic randomized controlled trial. BMJ. 2014;349:g5579.
76. Wakabayashi C, Hayashi K, Nagai K, Sakamoto N, Iwasaki Y. Effect of stamped reply envelopes and timing of newsletter delivery on response rates of mail survey: a randomised controlled trial in a prospective cohort study. BMJ Open. 2012;2(5):e001181.
77. Collins K, Reed M, Lifford K, Burton M, Edwards A, Ring A, et al. Bridging the age gap in breast cancer: evaluation of decision support interventions for older women with operable breast cancer: protocol for a cluster randomised controlled trial. BMJ Open. 2017;7(7):e015133.
78. Burchell AN, Allen VG, Grewal R, Macpherson PA, Rachlis A, Walmsley S, et al. Enhanced syphilis screening among HIV-positive men (ESSAHM): a study protocol for a clinic-randomized trial with stepped wedge design. Implementation Science. 2015;11(1).
79. Hestbaek L, Andersen ST, Skovgaard T, Olesen LG, Elmose M, Bleses D, et al. Influence of motor skills training on children's development evaluated in the Motor skills in PreSchool (MiPS) study-DK: study protocol for a randomized controlled trial, nested in a cohort study. Trials [Electronic Resource]. 2017;18(1):400.
80. Shields N, Willis C, Imms C, Prendergast LA, Watts JJ, Van Dorsselaer B, et al. FitSkills: protocol for a stepped wedge cluster randomised trial of a community-based exercise programme to increase participation among young people with disability. BMJ Open. 2020;10(7):e037153.
81. Solomon A, Kivipelto M, Molinuevo JL, Tom B, Ritchie CW. European Prevention of Alzheimer's Dementia Longitudinal Cohort Study (EPAD LCS): study protocol. BMJ Open. 2018;8(12):e021017.
82. er V, Verkooijen HM, Young-Afat DA, Burbach JP, Vulpen MV, Relton C, et al. The cohort multiple randomized controlled trial design: a valid and efficient alternative to pragmatic trials? International Journal of Epidemiology. 2017;46(1):96.
83. Mitchell N, Hewitt C, Adamson J, Parrott S, Torgerson D, Ekers D, et al. A randomised evaluation of CollAborative care and active surveillance for Screen-Positive EldeRs with sub-threshold depression (CASPER): study protocol for a randomized controlled trial. Trials [Electronic Resource]. 2011;12:225.
84. Peckham EJ, Relton C, Raw J, Walters C, Thomas K, Smith C. A protocol for a trial of homeopathic treatment for irritable bowel syndrome. BMC Complementary & Alternative Medicine. 2012;12:212.
85. Kwakkenbos L, Jewett LR, Baron M, Bartlett SJ, Furst D, Gottesman K, et al. The Scleroderma Patient-centered Intervention Network (SPIN) Cohort: protocol for a cohort multiple randomised controlled trial (cmRCT) design to support trials of psychosocial and rehabilitation interventions in a rare disease context. BMJ Open. 2013;3(8).
86. Uher R, Cumby J, MacKenzie LE, Morash-Conway J, Glover JM, Aylott A, et al. A familial risk enriched cohort as a platform for testing early interventions to prevent severe mental illness. BMC Psychiatry. 2014;14:344.
87. Viksveen P, Relton C. Depression treated by homeopaths: a study protocol for a pragmatic cohort multiple randomised controlled trial. Homeopathy: the Journal of the Faculty of Homeopathy. 2014;103(2):147.
88. Burbach JP, Verkooijen HM, Intven M, Kleijnen JP, Bosman ME, Raaymakers BW, et al. RandomizEd controlled trial for pre-operAtive dose-escaLation BOOST in locally advanced rectal cancer (RECTAL BOOST study): study protocol for a randomized controlled trial. Trials [Electronic Resource]. 2015;16:58.

For peer review only - http://bmjopen.bmj.com/site/about/guidelines.xhtml

89. Couwenberg AM, Burbach MJ, Smits AB, Van Vulpen M, Van Grevenstein WM, Noordzij PG, et al. The impact of retractor SPONGE-assisted laparoscopic surgery on duration of hospital stay and postoperative complications in patients with colorectal cancer (SPONGE trial): study protocol for a randomized controlled trial. Trials. 2016;17(1):132.
90. van der Velden JM, Verkooijen HM, Seravalli E, Hes J, Gerlich AS, Kasperts N, et al. Comparing conVEntional RadioTherapy with stereotactIC body radiotherapy in patients with spinAL metastases: study protocol for an randomized controlled trial following the cohort multiple randomized controlled trial design. BMC Cancer. 2016;16(1):909.
91. Lee H, Wiggers J, Kamper SJ, Williams A, Brien KMO, Hodder RK, et al. Mechanism evaluation of a lifestyle intervention for patients with musculoskeletal pain who are overweight or obese: protocol for a causal mediation analysis. BMJ Open. 2017;7(6):e014652.
92. Brunahl CA, Klotz SGR, Dybowski C, Riegel B, Gregorzik S, Tripp DA, et al. Combined Cognitive-Behavioural and Physiotherapeutic Therapy for Patients with Chronic Pelvic Pain Syndrome (COMBI-CPPS): study protocol for a controlled feasibility trial. Trials [Electronic Resource]. 2018;19(1):20.
93. Anderson BM, Dutton M, Day E, Jackson TA, Ferro CJ, Sharif A. Frailty Intervention Trial iN End-Stage patientS on haemodialysis (FITNESS): study protocol for a randomised controlled trial. Trials. 2018;19(1).
94. Relton C, Cathain AO, Nicholl J. A pilot 'cohort multiple randomised controlled trial' of treatment by a homeopath for women with menopausal hot flushes. Contemporary Clinical Trials. 2012;33(5):853.
95. Dascanio V, Birks Y, Clark L, Fairhurst C, MacPherson H, Torgerson DJ. Randomized cohort trial was shown to be feasible for evaluating treatments in low back pain. Journal of Clinical Epidemiology. 2014;67(8):940.
96. Cockayne S, Rodgers S, Green L, Fairhurst C, Adamson J, Scantlebury A, et al. Clinical effectiveness and cost-effectiveness of a multifaceted podiatry intervention for falls prevention in older people: a multicentre cohort randomised controlled trial (the REducing Falls with ORthoses and a Multifaceted podiatry intervention trial). Health Technology Assessment (Winchester, England). 2017;21(24):1.
97. Viksveen P, Relton C, Nicholl J. Depressed patients treated by homeopaths: a randomised controlled trial using the "cohort multiple randomised controlled trial" (cmRCT) design. Trials. 2017;18(1):299.
98. Brien KMO, Wiggers J, Williams A, Campbell E, Hodder RK, Wolfenden L, et al. Telephone-based weight loss support for patients with knee osteoarthritis: a pragmatic randomised controlled trial. Osteoarthritis & Cartilage. 2018;26(4):485.
99. Skaaby T, Jorgensen T, Linneberg A. A randomized general population study of the effects of repeated health checks on incident diabetes. Endocrine. 2018;60(1):122-8.
100. Williams A, Wiggers J, Brien KMO, Wolfenden L, Yoong SL, Hodder RK, et al. The effectiveness of a healthy lifestyle intervention, for chronic low back pain: a randomised controlled trial. Pain. 2018;2018 Mar 08.
101. Bibby AC, Torgerson DJ, Leach S, Lewis-White H, Maskell NA. Commentary: considerations for using the 'Trials within Cohorts' design in a clinical trial of an investigational medicinal product. Trials [Electronic Resource]. 2018;19(1):18.
102. Gal R, Monninkhof EM, Van Gils CH, Groenwold RHH, Elias SG, Van Den Bongard DHJG, et al. Effects of exercise in breast cancer patients: implications of the trials within cohorts (TwiCs) design in the UMBRELLA Fit trial. Breast Cancer Research and Treatment. 2021;190(1):89-101.
103. Vickers AJ, Vertosick EA, Carlsson SV, Ehdaie B, Kim SYH. Patient accrual and understanding of informed consent in a two-stage consent design. Clin Trials. 2021;18(3):377-82.
104. Mayo NE, Brouillette MJ, Fellows LK, Positive Brain Health Now I. Understanding and optimizing brain health in HIV now: protocol for a longitudinal cohort study with multiple randomized controlled trials. BMC Neurology. 2016;16:8.
105. Drew CJG, Quinn L, Hamana K, Williams-Thomas R, Marsh L, Dimitropoulou P, et al. Physical Activity and Exercise Outcomes in Huntington Disease (PACE-HD): Protocol for a 12-Month Trial Within

For peer review only - http://bmjopen.bmj.com/site/about/guidelines.xhtml

Cohort Evaluation of a Physical Activity Intervention in People With Huntington Disease. Physical Therapy. 2019;99(9):1201-10.
106. Heaven A, Brown L, Young J, Teale E, Hawkins R, Spilsbury K, et al. Community ageing research 75+ study (CARE75+): an experimental ageing and frailty research cohort. BMJ Open. 2019;9(3):e026744.
107. Wylie H, Cahill F, Santaolalla A, Moss CL, Enting D, Amery S, et al. Graham Roberts Study protocol: first 'trials within cohort study' for bladder cancer. BMJ Open. 2019;9(9):e029468.
108. Batenburg MCT, Van Den Bongard HJGD, Kleynen CE, Maarse W, Witkamp A, Ernst M, et al. Assessing the effect of hyperbaric oxygen therapy in breast cancer patients with late radiation toxicity (HONEY trial): a trial protocol using a trial within a cohort design. Trials. 2020;21(1).
109. Lee RZY, Yu J, Rawtaer I, Allen PF, Bao Z, Feng L, et al. CHI study: protocol for an observational cohort study on ageing and mental health in community-dwelling older adults. BMJ Open. 2020;10(5):e035003.
110. Newbold A, Warren FC, Taylor RS, Hulme C, Burnett S, Aas B, et al. Promotion of mental health in young adults via mobile phone app: study protocol of the ECoWeB (emotional competence for well-being in Young adults) cohort multiple randomised trials. BMC Psychiatry. 2020;20(1).
111. Schraa SJ, Van Rooijen KL, Van Der Kruijssen DEW, Rubio Alarcón C, Phallen J, Sausen M, et al. Circulating tumor DNA guided adjuvant chemotherapy in stage II colon cancer (MEDOCC-CrEATE): study protocol for a trial within a cohort study. BMC Cancer. 2020;20(1).
112. Brown L, Heaven A, Quinn C, Goodwin V, Chew-Graham C, Mahmood F, et al. Community Ageing Research 75+ (CARE75+) REMOTE study: a remote model of recruitment and assessment of the health, well-being and social circumstances of older people. BMJ Open. 2021;11(11):e048524.
113. Richters A, Meijer RP, Mehra N, Boormans JL, Van Der Heijden AG, Van Der Heijden MS, et al. Prospective bladder cancer infrastructure for experimental and observational research on bladder cancer: study protocol for the 'trials within cohorts' study ProBCI. BMJ Open. 2021;11(5):e047256.
114. Holt H, Relton C, Talaei M, Symons J, Davies MR, Jolliffe DA, et al. Cohort Profile: Longitudinal population-based study of COVID-19 in UK adults (COVIDENCE UK). International Journal of Epidemiology. 2022.
115. Witlox L, Hiensch AE, Velthuis MJ, Steins Bisschop CN, Los M, Erdkamp FLG, et al. Four-year effects of exercise on fatigue and physical activity in patients with cancer. BMC Medicine. 2018;16(1).
116. Couwenberg AM, Burbach JPM, Berbee M, Lacle MM, Arensman R, Raicu MG, et al. Efficacy of Dose-Escalated Chemoradiation on Complete Tumor Response in Patients with Locally Advanced Rectal Cancer (RECTAL-BOOST): A Phase 2 Randomized Controlled Trial. International Journal of Radiation Oncology*Biology*Physics. 2020;108(4):1008-18.
117. Pielkenrood BJ, Van Der Velden JM, Van Der Linden YM, Bartels MMT, Kasperts N, Verhoeff JJC, et al. Pain Response After Stereotactic Body Radiation Therapy Versus Conventional Radiation Therapy in Patients With Bone Metastases—A Phase 2 Randomized Controlled Trial Within a Prospective Cohort. International Journal of Radiation Oncology*Biology*Physics. 2020.
118. Grimmer K, Luker J, Beaton K, Kumar S, Crockett A, Price K. TRialing individualized interventions to prevent functional decline in at-risk older adults (TRIIFL): study protocol for a randomized controlled trial nested in a longitudinal observational study. Trials [Electronic Resource]. 2013;14:266.
119. Merat S, Poustchi H, Hemming K, Jafari E, Radmard AR, Nateghi A, et al. PolyPill for Prevention of Cardiovascular Disease in an Urban Iranian Population with Special Focus on Nonalcoholic Steatohepatitis: A Pragmatic Randomized Controlled Trial within a Cohort (PolyIran - Liver) - Study Protocol. Archives of Iranian Medicine. 2015;18(8):515.
120. Neves JB, Cullen D, Grant L, Walkden M, Bandula S, Patki P, et al. Protocol for a feasibility study of a cohort embedded randomised controlled trial comparingNEphronSparingTreatment (NEST) for small renal masses. BMJ Open. 2019;9(6):e030965.
121. Desai M. Recruitment and retention of participants in clinical studies: Critical issues and challenges. Perspect Clin Res. 2020;11(2):51-3.

For peer review only - http://bmjopen.bmj.com/site/about/guidelines.xhtml

122. Young-Afat DA, van Gils CH, van den Bongard H, Verkooijen HM, Group US. The Utrecht cohort for Multiple BREast cancer intervention studies and Long-term evaLuAtion (UMBRELLA): objectives, design, and baseline results. Breast Cancer Res Treat. 2017;164(2):445-50.

123. Coebergh van den Braak RRJ, Rijssen LBv, Kleef JJv, Vink GR, Berbee M, Henegouwen MIvB, et al. Nationwide comprehensive gastro-intestinal cancer cohorts: the 3P initiative. Acta Oncologica. 2018;57(2):195.

124. er V, Verkooijen HM, Seravalli E, Hes J, Gerlich AS, Kasperts N, et al. Comparing conVEntional RadioTherapy with stereotactIC body radiotherapy in patients with spinAL metastases: study protocol for an randomized controlled trial following the cohort multiple randomized controlled trial design. BMC Cancer. 2016;16(1):909.

For peer review only

For peer review only - http://bmjopen.bmj.com/site/about/guidelines.xhtml

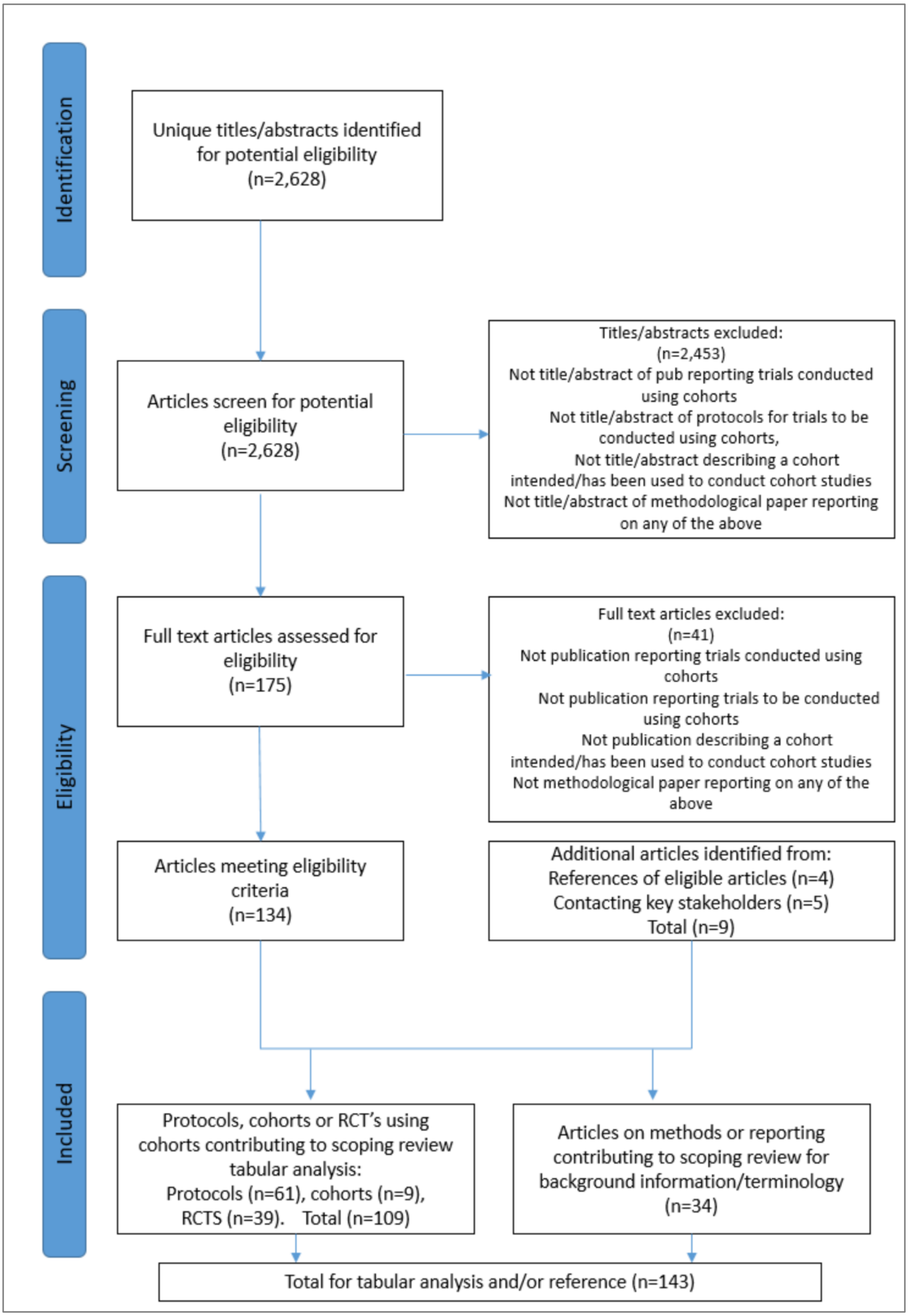
Identification
Unique titles/abstracts identified for potential eligibility (n=2,628)
Screening
Articles screen for potential eligibility (n=2,628)
Titles/abstracts excluded: (n=2,453) Not title/abstract of pub reporting trials conducted using cohorts Not title/abstract of protocols for trials to be conducted using cohorts, Not title/abstract describing a cohort intended/has been used to conduct cohort studies Not title/abstract of methodological paper reporting on any of the above
Eligibility
Full text articles assessed for eligibility (n=175)
Full text articles excluded: (n=41) Not publication reporting trials conducted using cohorts Not publication reporting trials to be conducted using cohorts Not publication describing a cohort intended/has been used to conduct cohort studies Not methodological paper reporting on any of the above
Articles meeting eligibility criteria (n=134)
Additional articles identified from: References of eligible articles (n=4) Contacting key stakeholders (n=5) Total (n=9)
Included
Protocols, cohorts or RCT's using cohorts contributing to scoping review tabular analysis: Protocols (n=61), cohorts (n=9), RCTS (n=39). Total (n=109)
Articles on methods or reporting contributing to scoping review for background information/terminology (n=34)
Total for tabular analysis and/or reference (n=143)

Electronic search strategies

Adapted from the published protocol(24). Searches were run in both MEDLINE and Cochrane Methodology Register simultaneously. As an example, in the cohort search, lines 1-11 are the MEDLINE search and lines 12-15 are tailored for the Cochrane Methodology Register. The final lines of each search isolate the records from each database, combine them so duplicate records can be removed, then isolate the remaining records so they can be downloaded and imported into Reference Manager using customized import filters.

Searches for RCTs embedded in Cohorts

1. (cohort adj5 (randomi#ed adj5 trial*)).ab,kf,ti.

2. (cohort adj5 RCT*).ab,kf,ti.

3. (cohort adj5 controlled trial*).ab,kf,ti.

4. (cmRCT or Cohort Multiple Randomised Controlled Trial*).ab,kf,ti.

5. or/1-4

6. cohort.af.

7. (embed* adj8 randomi#ed).ab,kf,ti.

8. (embed* adj8 RCT*).ab,kf,ti.

9. (embed* adj8 controlled trial*).ab,kf,ti.

10. or/7-9

11. 6 and 10

12. (pragmatic adj5 RCT*).ab,kf,ti.

13. (pragmatic adj5 randomi#ed).ab,kf,ti.

14. (pragmatic adj5 controlled trial*).ab,kf,ti.

15. or/12-14

16. 6 and 15

17. 5 or 11 or 16

18. (meta analy* or metaanaly* or metanaly* or systematic review*).af.

19. 17 not 18

20. limit 19 to yr="2007 - 2018"

21. ((Cohort* and (random* or RCT)) or cmRCT).ti,ab,kw.

22. limit 21 to yr="2007 - 2018"

23. 20 use medall

24. 22 use clcmr

For peer review only

25. 23 or 24

26. remove duplicates from 25

27. 26 use medall

28. 26 use clcmr

For peer review only

Inclusion/Exclusion criteria for studies

Adapted from protocol for a scoping review to support development of a CONSORT extension for randomised controlled trials using cohorts and routinely collected health data(24).

Exclude: The publication is NOT reporting trials conducted using cohorts, protocols for RCTs that will be conducted using cohorts, describing a cohort intended to be used or has been used to conduct RCTs or methodological papers describing the use of cohorts. If the study involves non-human subjects it will be excluded.

Include: The publication describes an RCT in which the cohort is used or will be used to identify trial participants. The publication describes a cohort used to identify trial participants.

Include: The publication describes an RCT in which the cohort is used or will be used to ascertain health outcomes. The publication describes a cohort used to ascertain health outcomes.

Include: The methodological papers describe the use of cohorts for RCTs.

For peer review only

Preferred Reporting Items for Systematic reviews and Meta-Analyses extension for Scoping Reviews (PRISMA-ScR) Checklist

SECTION	ITEM	PRISMA-ScR CHECKLIST ITEM	REPORTED ON PAGE #
**TITLE**			
Title	1	Identify the report as a scoping review.	
**ABSTRACT**			
Structured summary	2	Provide a structured summary that includes (as applicable): background, objectives, eligibility criteria, sources of evidence, charting methods, results, and conclusions that relate to the review questions and objectives.	
**INTRODUCTION**			
Rationale	3	Describe the rationale for the review in the context of what is already known. Explain why the review questions/objectives lend themselves to a scoping review approach.	
Objectives	4	Provide an explicit statement of the questions and objectives being addressed with reference to their key elements (e.g., population or participants, concepts, and context) or other relevant key elements used to conceptualize the review questions and/or objectives.	
**METHODS**			
Protocol and registration	5	Indicate whether a review protocol exists; state if and where it can be accessed (e.g., a Web address); and if available, provide registration information, including the registration number.	
Eligibility criteria	6	Specify characteristics of the sources of evidence used as eligibility criteria (e.g., years considered, language, and publication status), and provide a rationale.	
Information sources*	7	Describe all information sources in the search (e.g., databases with dates of coverage and contact with authors to identify additional sources), as well as the date the most recent search was executed.	
Search	8	Present the full electronic search strategy for at least 1 database, including any limits used, such that it could be repeated.	
Selection of sources of evidence†	9	State the process for selecting sources of evidence (i.e., screening and eligibility) included in the scoping review.	
Data charting process‡	10	Describe the methods of charting data from the included sources of evidence (e.g., calibrated forms or forms that have been tested by the team before their use, and whether data charting was done independently or in duplicate) and any processes for obtaining and confirming data from investigators.	
Data items	11	List and define all variables for which data were sought and any assumptions and simplifications made.	
Critical appraisal of individual sources of evidence§	12	If done, provide a rationale for conducting a critical appraisal of included sources of evidence; describe the methods used and how this information was used in any data synthesis (if appropriate).	
Synthesis of results	13	Describe the methods of handling and summarizing the data that were charted.	

For peer review only

SECTION	ITEM	PRISMA-ScR CHECKLIST ITEM	REPORTED ON PAGE #
**RESULTS**			
Selection of sources of evidence	14	Give numbers of sources of evidence screened, assessed for eligibility, and included in the review, with reasons for exclusions at each stage, ideally using a flow diagram.	
Characteristics of sources of evidence	15	For each source of evidence, present characteristics for which data were charted and provide the citations.	
Critical appraisal within sources of evidence	16	If done, present data on critical appraisal of included sources of evidence (see item 12).	
Results of individual sources of evidence	17	For each included source of evidence, present the relevant data that were charted that relate to the review questions and objectives.	
Synthesis of results	18	Summarize and/or present the charting results as they relate to the review questions and objectives.	
**DISCUSSION**			
Summary of evidence	19	Summarize the main results (including an overview of concepts, themes, and types of evidence available), link to the review questions and objectives, and consider the relevance to key groups.	
Limitations	20	Discuss the limitations of the scoping review process.	
Conclusions	21	Provide a general interpretation of the results with respect to the review questions and objectives, as well as potential implications and/or next steps.	
**FUNDING**			
Funding	22	Describe sources of funding for the included sources of evidence, as well as sources of funding for the scoping review. Describe the role of the funders of the scoping review.	

JBI = Joanna Briggs Institute; PRISMA-ScR = Preferred Reporting Items for Systematic reviews and Meta-Analyses extension for Scoping Reviews.

* Where *sources of evidence* (see second footnote) are compiled from, such as bibliographic databases, social media platforms, and Web sites.

† A more inclusive/heterogeneous term used to account for the different types of evidence or data sources (e.g., quantitative and/or qualitative research, expert opinion, and policy documents) that may be eligible in a scoping review as opposed to only studies. This is not to be confused with *information sources* (see first footnote).

‡ The frameworks by Arksey and O'Malley (6) and Levac and colleagues (7) and the JBI guidance (4, 5) refer to the process of data extraction in a scoping review as data charting.

§ The process of systematically examining research evidence to assess its validity, results, and relevance before using it to inform a decision. This term is used for items 12 and 19 instead of "risk of bias" (which is more applicable to systematic reviews of interventions) to include and acknowledge the various sources of evidence that may be used in a scoping review (e.g., quantitative and/or qualitative research, expert opinion, and policy document).

From: Tricco AC, Lillie E, Zarin W, O'Brien KK, Colquhoun H, Levac D, et al. PRISMA Extension for Scoping Reviews (PRISMAScR): Checklist and Explanation. Ann Intern Med. 2018;169:467–473. doi: 10.7326/M18-0850.

For peer review only - http://bmjopen.bmj.com/site/about/guidelines.xhtml
